# Divergence of dim-light vision among bats (order: Chiroptera) as estimated by molecular and electrophysiological methods

**DOI:** 10.1038/srep11531

**Published:** 2015-06-23

**Authors:** He-Qun Liu, Jing-Kuan Wei, Bo Li, Ming-Shan Wang, Rui-Qi Wu, Joshua D. Rizak, Li Zhong, Lu Wang, Fu-Qiang Xu, Yong-Yi Shen, Xin-Tian Hu, Ya-Ping Zhang

**Affiliations:** 1 State Key Laboratory of Genetic Resources and Evolution, Yunnan Laboratory of Molecular Biology of Domestic Animals, Kunming Institute of Zoology, Chinese Academy of Sciences, Kunming, 650223, China; 2 Key Laboratory of Animal Models and Human Disease Mechanisms, Kunming Institute of Zoology, Chinese Academy of Sciences, Kunming, 650223, China; 3 Joint Influenza Research Centre (SUMC/HKU), Shantou University Medical College, Shantou, 515041, China; 4 State Key Laboratory of Magnetic Resonance and Atomic and Molecular Physics, and Key Laboratory of Magnetic Resonance in Biological Systems, Wuhan Institute of Physics and Mathematics, Chinese Academy of Sciences, Wuhan, 430071, China; 5 Laboratory for Conservation and Utilization of Bio-resource, Yunnan University, Kunming, 650091, China; 6 Kunming College of Life Science, University of the Chinese Academy of Sciences, Kunming, 650204, China; 7 University of the Chinese Academy of Sciences, Beijing, China

## Abstract

Dim-light vision is present in all bats, but is divergent among species. Old-World fruit bats (Pteropodidae) have fully developed eyes; the eyes of insectivorous bats are generally degraded, and these bats rely on well-developed echolocation. An exception is the Emballonuridae, which are capable of laryngeal echolocation but prefer to use vision for navigation and have normal eyes. In this study, integrated methods, comprising manganese-enhanced magnetic resonance imaging (MEMRI), f-VEP and RNA-seq, were utilized to verify the divergence. The results of MEMRI showed that Pteropodidae bats have a much larger superior colliculus (SC)/ inferior colliculus (IC) volume ratio (3:1) than insectivorous bats (1:7). Furthermore, the absolute visual thresholds (log cd/m^2^•s) of Pteropodidae (−6.30 and −6.37) and Emballonuridae (−3.71) bats were lower than those of other insectivorous bats (−1.90). Finally, genes related to the visual pathway showed signs of positive selection, convergent evolution, upregulation and similar gene expression patterns in Pteropodidae and Emballonuridae bats. Different results imply that Pteropodidae and Emballonuridae bats have more developed vision than the insectivorous bats and suggest that further research on bat behavior is warranted.

Briefly, visual processing in the visual system is believed to occur in two steps: first, optic signals are transduced from the environment into neuronal signals in the retina via cis/trans retinal isomerization, cGMP-gated signaling, synaptic transmission, and action potentials^1^,^2^,^3^,^4^,^5^; next, the neuronal signals are transformed and integrated into the visual pathway and eventually rebuilt into a pattern in the brain^6^. Different visual systems have evolved in many vertebrates to adapt to various light environments^7^,^8^. For example, the diurnal species *Anolis carolinensis* has pure-cone retinas because cone photoreceptors distinguish color very well^8^; and the nocturnal animals have adopted certain strategies to enhance visual reliability in dim light, including improving the capture of photons in the retina^9^,^10^, like *Gekko gecko*, that has pure-rod retinas^8^. Such adaptive changes in the retina suggested that these animals might have specific structures through the visual pathway to process differential visual information^11^, although little previous experimental research has verified this model.

The superior colliculus (SC) and inferior colliculus (IC), located in the dorsal midbrain, have well-established functions in visual and auditory processing respectively. The SC, particularly in non-human primates^12^,^13^, plays a vital role in the neural control of saccadic eye movements, which can help them accurately fixate on an object suddenly appearing in the visual field. The IC primarily participates in detecting and analyzing auditory information and then codes that information for spatial localization^14^,^15^. Therefore, differences in the relative sizes of the SC and IC likely reflect different degrees of utilization of vision and hearing.

Bats are nocturnal and most species of bats have functional eyes^16^,^17^,^18^, but vision is variable across species. Old-World fruit bats (Pteropodidae, the only family of megabats) have developed vision and relatively large eyes^19^, whereas the insectivorous bats (microbats) have developed laryngeal echolocation and less developed eyes^20^,^21^, they use vision in long-distance navigation and distinguishing day from night and possess big corneal surfaces and lenses relative to the size of their eye^20^. Besides, the megabats have an advanced retinotectal pathway similar to that in primates, whereas microbats have a retinotectal pathway analogous to all vertebrates except primates^22^ and though it is not known whether the ancestors of bats used echolocation or vision, living Pteropodidae have enlarged eye orbits and insectivorous bats have enlarged cochleae^23^,^24^. Interestingly, the Emballonuridae bats, belongs to insectivorous bats, are an exception. These bats can use laryngeal echolocation, they also have larger eyes and appear to rely more heavily on visual sight than other insectivorous bats^20^,^21^. Furthermore, Emballonuridae possess relatively larger SC than Old-World fruit bats^25^.

As described above, we hypothesized that certain anatomical structures associated with the visual pathway, visual capacity, and genes participating in the visual pathway have diverged within Chiroptera. To test this hypothesis, the present study used MEMRI (manganese-enhanced magnetic resonance imaging), which produces images with high spatial resolution for understanding anatomy and has been applied to detect neuronal activity *in vivo* and explore neuronal structures^26^,^27^, to measure the brain anatomy of selected species of bats. The volumes of the SC and IC from each species were calculated. Higher SC/IC volume ratios were found in Pteropodidae bats (3:1) than insectivorous bats (1:7), indicating that Pteropodidae are more apt to use vision for navigation than insectivorous bats. Flash-visual evoked potentials (f-VEP), an electrophysiological method, were used to measure the absolute visual threshold^28^,^29^ of four species of bats. As predicted, the lowest absolute visual thresholds were found in Pteropodidae (−6.30 and −6.37, log cd/m^2^•s), followed by Emballonuridae (−3.71) and then insectivorous bats (−1.90). Finally, to better understand the potential genetic basis of this divergence of vision in bats, which was likely based on either a single gene or a few genes^30^,^31^, five transcriptomes from five species of bats covering major groups of bats were sequenced. Positive selection and upregulation of the genes involved in the visual pathway were detected in the Pteropodidae and Emballonuridae compared with the insectivorous bats, and similar patterns of gene expression were found in Pteropodidae and Emballonuridae. Taken together, our study used a combination of imaging, electrophysiology, and RNA-seq methods to elaborate on the visual divergence in bats and showed that Pteropodidae and Emballonuridae rely more on vision than insectivorous bats, suggesting that the behavior of bats requires additional attention.

## Results

### The visual pathway in bats

**Figure 1:**
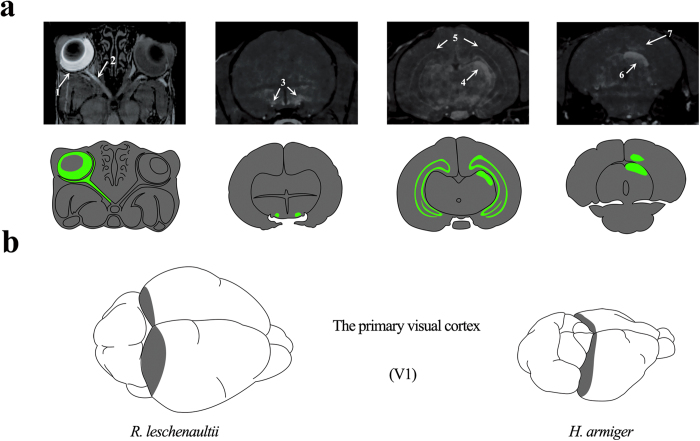
MEMRI of *R. leschenaultii* and the location of V1 in bats. **a**). MRI of the Mn^2+^-enhanced visual pathway performed 24 hours after the intravitreal injection of Mn^2+^ into the left eye of *R. leschenaultii*. Annotations: retina (1), optic nerve (2), optic chiasm (3), lateral geniculate nucleus (4), hippocampus (5), superior colliculus (6) and primary visual cortex (7). **b**). The primary visual cortices of *R. leschenaultii* and *H. armiger.*

In total, seven species of bats, including the Pteropodidae bats (*Rousettus leschenaultii*, *Cynopterus sphinx* and *Eonycteris spelaea*), the Emballonuridae (*Taphozous melanopogon*) and the insectivorous bats (*Hipposideros armiger*, *Rhinolophus affinis* and *Myotis laniger*), were used in this study. Initially, the MRI experiment was used in this study to examine the brain structural differences in the visual systems of Pteropodidae bats and insectivorous bats. MRIs were conducted 24 hours after Mn^2+^ injections into the left eye of *R. leschenaultii* (Pteropodidae bats). Clear delineation of the components in the visual pathway was visible in the post-injection T1-weighted images. MRI of *R. leschenaultii* depicted relatively clear Mn^2+^ enhancement throughout the visual pathway (Fig. 1a). The left retina (arrow 1) and optic nerve (arrow 2) were considerably enhanced compared with the same structures on the contralateral side, which did not receive Mn^2+^ injection. At the optic chiasm (arrow 3), most axons crossed to the contralateral side, where the projections continued to the lateral geniculate nucleus (arrow 4) and SC (arrow 6). The contralateral primary visual cortex (arrow 7) also showed enhanced signals, albeit to a lesser extent. In addition to the central visual system, other brain structures, such as the bilateral hippocampus (arrow 5), were also partially enhanced by Mn^2+^, indicating brain activity over the last 24 hours. A comparative schematic between *R. leschenaultii* and *H. armiger* brains is shown in Fig. 1b (developed in Photoshop CS5) to highlight the respective primary visual cortex.

### Comparison of the SC and IC in Pteropodidae vs. insectivorous bats

**Figure 2:**
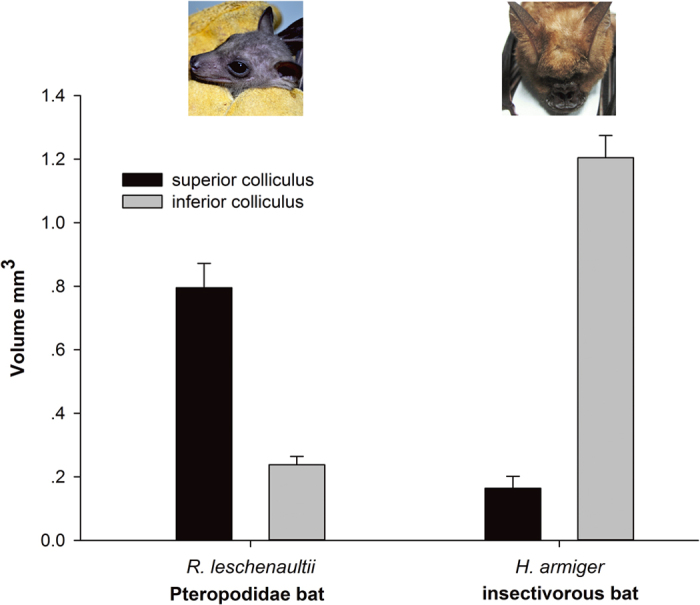
Comparison of SC and IC volumes between Pteropodidae and insectivorous bats. SC and IC volume was calculated from the area of the SC/IC in each sequence from the MRI multiplied by the depth of each sequence. The SC/IC volume ratio is approximately 3:1 in *R. leschenaultii* and approximately 1:7 in *H. armiger*, n = 3. (The photograph of *R. leschenaultii* was taken by H.Q.L., and the photograph of *H. armiger* was obtained from Xu-Dong Zhao).

MRI was performed 24 hours after the intraperitoneal injection of Mn^2+^ in 3 *R. leschenaultii* (Pteropodidae) and 3 *H. armiger* (insectivorous) bats, respectively. Then, SC and IC volume (the area of the SC/IC in each sequence from the MRI multiplied by the depth of each sequence) was calculated (details in Fig. 2, SigmaPlot Suite V 12.5). *R. leschenaultii* had a large SC and small IC, with an SC/IC volume ratio of approximately 3:1. By contrast, *H. armiger* had a large IC and small SC (SC/IC ratio was 1:7). Higher ratios of SC volume to IC volume in Pteropodidae may indicate that these bats are better able to control the eye movements and more likely to employ vision than insectivorous bats.

### Assessment of absolute visual thresholds

An electrophysiological method was then used to measure the divergence of visual function among bats. Four bat species, including Pteropodidae (*R. leschenaultii* and *C. sphinx*), insectivorous (*R. affinis*) and Emballonuridae (*T. melanopogon*) bats, were evaluated for their visual thresholds using the flash-visual evoked potential (f-VEP) method.

**Figure 3:**
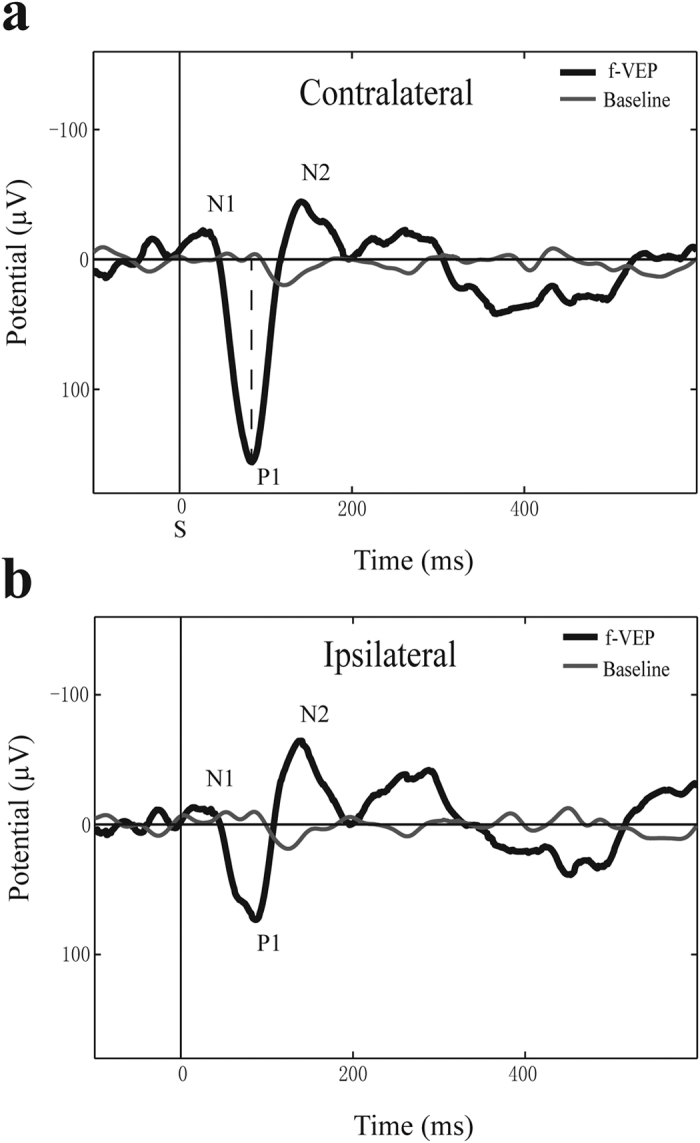
Representative flash VEP waveforms from *C. sphinx*. **a**). The contralateral cortex f-VEP. **b**). The ipsilateral cortex f-VEP. Annotations: the N1-P1-N2 peak structure; vertical dashed line represents the position of the P1 amplitude measure; S indicates flash stimulus.

Waveforms of evoked potentials vary between animal species due to differences in brain structure. Therefore, the waveforms were manipulated using methods similar to those in previous studies by extracting waveform components and a common index to make them comparable^32^. An example of the f-VEP waveforms from our results measured contralaterally in *C. sphinx* (Pteropodidae bat) was shown in Fig. 3a. There were three consistent wave components in f-VEP waveforms at early response time (≤500 ms) (Fig. 3a), which were sequentially labeled as the first negative wave (N1, 40–60 ms), first positive wave (P1, 90–120 ms) and second negative wave (N2, 140–200 ms). This N-P-N structure was extracted as a common index to illustrate waveform changes in response to the intensities of gradually decreasing flash stimuli. In addition, an evoked waveform of the ipsilateral cortex of *C. sphinx* is shown in Fig. 3b. Here, the waveforms of the contralateral cortex were higher in amplitude than those in the ipsilateral cortex, which corresponds with the results obtained in the MEMRI experiments with unilateral injection of Mn^2+^.

**Figure 4:**
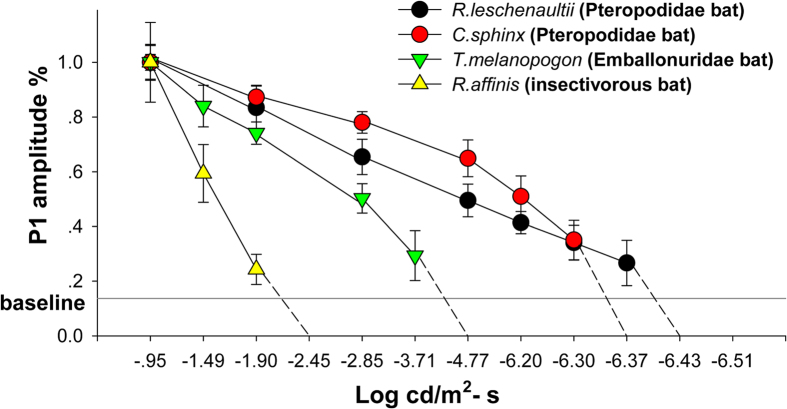
Estimates of visual threshold in 4 bat species. A gradual decrease in VEP P1 amplitude (%) was observed with decreasing flash intensity. Baseline: mean value of the highest P1 peak amplitude (%) compared with the baseline for 4 species of bats, mean ± SD.

The f-VEP results in the four bats are shown in Fig. S1. The range from approximately −0.49 to −6.51 (log cd/m^2^•s) at the 12 light intensity levels was used to make a more accurate measurement. A decreasing trend of N-P-N amplitude appeared as the flash intensities decreased. The percentages of P1 amplitude (the highest peaks in the N-P-N structure) were used to compare the declining trend in each bat in response to specific light intensities (Fig. 4, SigmaPlot Suite V 12.5). The absolute visual threshold was defined as the lowest flash intensity that produced an N-P-N structure distinguishable from baseline; i.e., when the light intensity was lower than the absolute visual threshold, the bat could not see. The absolute visual threshold intensity for each species was −1.90 (log cd/m^2^•s) for *R. affinis*, −3.71 (log cd/m^2^•s) for *T. melanopogon* and −6.30 and −6.37 (log cd/m^2^•s) for *C. sphinx* and *R. leschenaultii*, respectively. The dim-light visual perception boundary of insectivorous bats, as measured by f-VEP, occurred at a higher terminal photic intensity than that of Pteropodidae, indicating weaker dim-light vision in insectivorous bats. Emballonuridae had intermediate visual ability in terms of light intensity.

In addition, Pteropodidae and insectivorous bats also had a divergent pattern in respect to the latency of the N-P-N structure wave components. The latency was measured as the time interval from the stimulus (S) to the wave peaks (Fig. 3a). *R. leschenaultii* and *C. sphinx* (Pteropodidae bats) had similar latency in all three waves, suggesting similar visual pathways in Pteropodidae bats. However, the f-VEP waveforms of *R. affinis* (insectivorous bats) were different and did not show an obvious N1 peak. The latencies of the other two peaks (P1 & N2) were approximately 20 ms slower than in the other bat species (Table. 1).

### RNA-seq and *de novo* assembly

The f-VEP results indicated that different visual capacities exist in bats, but the molecular mechanism, particularly the molecular mechanism of photoreception, remained to be elaborated. Here, the eye transcriptomes of five species of bat, including Pteropodidae (*E. spelaea* and *C. sphinx*), insectivorous (*H. armiger* and *M. laniger*) and Emballonuridae (*T. melanopogon*) bats, were sequenced using automated sequencers. The systems produced an average of 68.9 million (6.72 Gb) raw cDNA reads for each species. Of those reads, 60.1 million (5.15 Gb) high-quality pair-end reads were obtained for every species and used for *de novo* assembly (Table S1). The average N50 statistic for all samples was approximately 1,726 bp (contig length > 200 bp). The scale of the longest contigs in the 5 species ranged from 17,727 bp to 25,539 bp. Approximately 11,172 one-to-one orthologous genes were detected in each sample (Table S2).

### Positive selection analyses

**Figure 5:**
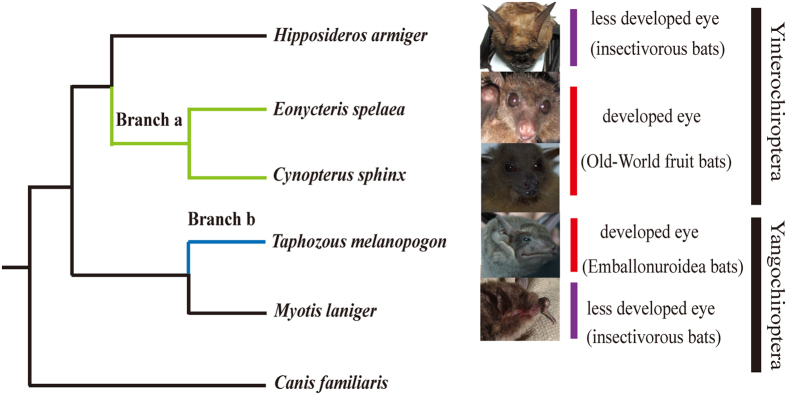
Phylogenetic tree used in the analysis of positive selection and parallel evolution. Branch a (green line) represents the branch leading to the ancestor of Pteropodidae. Branch b (blue line) represents the Emballonuridae bat. Each branch was defined as a foreground branch. The study of parallel evolution focused on either branch a or b (the photographs of *C. sphinx* and *M. laniger* were taken by H.Q.L., and photographs of *H. armiger*, *E. spelaea* and *T. melanopogon* were obtained from Xu-Dong Zhao).

In total, 7,002 one-to-one orthologous genes were used in the multiple alignments. After trimming, 5,688 genes remained and were used for positive selection. Positively selected genes (PS genes, test 2) and positively selected sites (PSSs, BEB posterior probability ≥ 0.9) were explored using the branch-site model. First, the ancestor branch of Pteropodidae (branch a in Fig. 5) was set as the foreground branch, which identified 112 positively selected genes (*P *≤ 0.05). Next, 95 PSSs were found from among 67 PS genes. Second, Emballonuridae (branch b in Fig. 5) was set as the foreground branch, which identified 160 positively selected genes (*P *≤ 0.05). Next, 162 PSSs were found from 103 PS genes. After a FDR correction, 39 and 81 PS genes remained in branches a and b, respectively (detailed in Tables S3 and S4).

### Analyses of parallel and convergent evolution

Ancestor sequences of the internal nodes were reconstructed by PAML. Branches a and b (branches corresponding to Pteropodidae and Emballonuridae, respectively; Fig. 5) were analyzed to identify genes that underwent parallel or convergent evolution. These two branches represent two lineages that have larger eyes and better vision. In total, 330 genes (409 sites) had parallel amino acid changes (Table S5), and no gene explored underwent convergent amino acid changes. Genes that displayed parallel evolution were enriched in terms of gene ontology (GO) for the sensory perception of light (GO: 0050953, *P *= 0.0110) and visual perception (GO: 0007601, *P *= 0.0110). In particular, *CRX* had a parallel change at amino acid position 133 (P133A), which was consistent with the previous study^31^. In total, 20 genes, including 3 genes encoding uncharacterized proteins (*PWP1, COG1, ATP8A2, ATP1B3, MYOF, PINX1, ECI2, IFIT1, ACSL5, OTUD7B, DTX3L, SRGN, C1R, TAPBPL, FKBP4, TTLL5 and COL8A1*), and 20 genes, including 1 gene encoding an uncharacterized protein (*TAB2, ACO2, GLDC, STRN, RP1, CRYL1, FDFT1, HEXB, ECI2, ACSL5, DCAF11, AP4M1, RHNO1, EIF2AK1, VSIG4, CALCOCO2, SKP2, MPV17* and *SCP2*), were found to have signals of both positive selection and parallel amino acid changes in branches a and b, respectively. Of these, ECI2 and ACSL5 had positive selection in branches a and b.

Then, 10 one-to-one ortholog coding sequences were retrieved from additional species (horse, cow, dolphin, rat, mouse, human, gorilla, chimpanzee, macaque and orangutan) in Ensembl 70 to detect whether these sites exhibited signs of conservation. The parallel amino acid changes of 6 genes, including 1 gene encoding an uncharacterized protein (*ACO2*, *COG1*, *STRN*, *ATP8A2* and *MYOF*), were conserved. In addition, the *ACO2* positive selection site that was investigated was also conserved.

### Expression profiles and principal components analysis (PCA)

The five species of bats were divided into three groups according to their divergent reliance on vision, and three comparisons were performed as follows: comparison A: *C. sphinx* and *E. spelaea* (Pteropodidae bats with well-developed eyes and without echolocation) *vs. H. armiger* and *M. laniger* (less developed eyes and echolocation); comparison B: *T. melanopogon* (normal eyes and echolocation) *vs. H. armiger* and *M. laniger* (less developed eyes and echolocation); and comparison C: *C. sphinx* and *E. spelaea* (developed eyes and no echolocation) *vs. T. melanopogon* (normal eyes and echolocation).

In comparison A, 2,464 genes had significantly higher gene expression in the Pteropodidae family. These genes were enriched in terms of their responses to retinoic acid (GO: 0032526, *P *= 0.007) and vitamin A (GO: 0033189, *P *= 0.039). In comparison B, 1,947 genes had significantly higher expression in Emballonuridae. These genes were enriched in terms of lens development in a camera-type eye (GO: 0002088, *P *= 2.31E−04), camera-type eye development (GO: 0043010, *P *= 0.002), response to light stimulus (GO: 0009416, *P *= 0.006), eye development (GO: 0001654, *P *= 0.006), camera-type eye morphogenesis (GO: 0048593, *P *= 0.011), lens morphogenesis in a camera-type eye (GO: 0002089, *P *= 0.019), eye morphogenesis (GO: 0048592, *P *= 0.019), visual behavior (GO: 0007632, *P *= 0.031) and the retinoic acid receptor signaling pathway (GO: 0048384, *P *= 0.035). In comparison C, 2,885 genes were differentially expressed. However, these genes were not enriched in any of the GO terms associated with vision.

We identified 914 genes showing higher expression in Pteropodidae bats and *T. melanopogon* than *H. armiger* and *M. laniger*, but no significant differences in expression were observed between the Pteropodidae bats and *T. melanopogon*. These genes were enriched in terms of camera-type eye development (GO: 0043010, *P = 0.005*) and eye development (GO: 0001654, *P = 0.027*).

**Figure 6:**
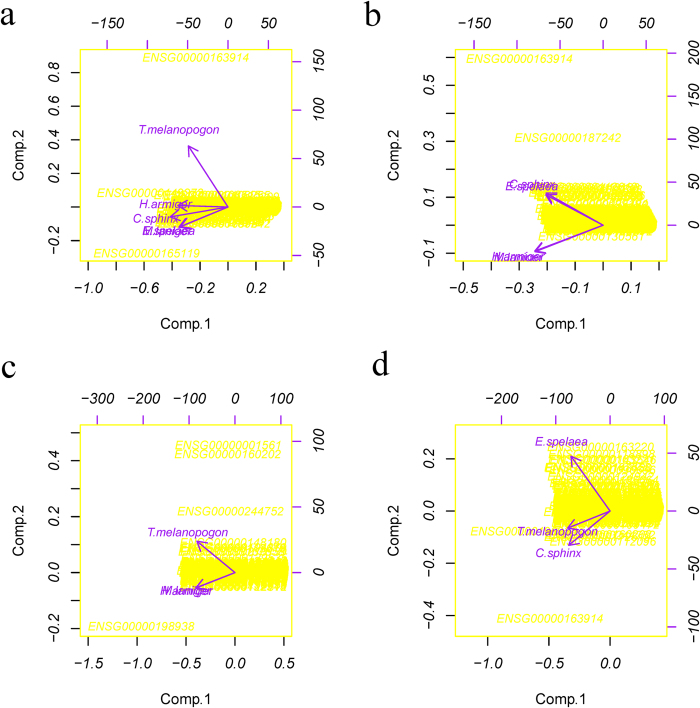
Results of the principal components analysis. **a**). PCA of the gene expression patterns in five species of bats. **b**). PCA of the gene expression patterns in comparison A: the Pteropodidae bats *vs.* the insectivorous bats (*E. spelaea* and *C. sphinx vs. H. armiger* and *M. laniger*). **c**). PCA of the gene expression patterns in comparison B: the Emballonuridae bats vs. the insectivorous bats (*T. melanopogon vs. H. armiger* and *M. laniger*). d). PCA of the gene expression patterns in Comparison C: the Pteropodidae bats *vs.* the Emballonuridae bats (*E. spelaea* and *C. sphinx vs. T. melanopogon*). Each arrow represents a single species. Along each principal component, the same direction indicates a similar pattern, and the shorter distances indicate more similar gene expression patterns. The left and bottom axes are showing the normalized principal component scores; the top and right axes are showing the loadings.

The PCA was conducted among all five species of bats and the three comparison groups (details in Fig. 6). When comparing all five species of bats, the pattern of gene expression in *T. melanopogon* was different from the other four species because they showed the reverse direction in terms of the second principal component, and the gene *RHO* (*Rh*1, ENSG00000163914) exhibited the highest expression in *T. melanopogon* (Fig. 6A). While addressing each comparison, the Pteropodidae bats (*E. spelaea* and *C. sphinx*) clustered together, and the insectivorous bats (*H. armiger* and *M. laniger*) clustered together (Fig. 6b). In addition, *T. melanopogon* showed a different gene expression pattern than the insectivorous bats (*H. armiger* and *M. laniger*), which clustered together (Fig. 6c). However, the gene expression pattern of *T. melanopogon* clustered between *E. spelaea* and *C. sphinx*, the Pteropodidae bats (Fig. 6d).

## Discussion

In this work, anatomical differences were observed, such as the larger SC in the Pteropodidae bats and the larger IC in the insectivorous bats, as well as more robust visual capacity in the Pteropodidae and Emballonuridae bats than in the insectivorous bats. Finally, genes participating in the visual pathway exhibited signs of positive selection and parallel evolution, and the Pteropodidae and Emballonuridae bats had similar gene expression patterns. All of these results demonstrate that Pteropodidae bats and Emballonuridae bats developed better visual ability than other insectivorous bats. Furthermore, it implied that attention should be pay on the function and behavior in term of research on the adaptive evolution and our research may serve as a model.

Compared with the lateral geniculate nucleus, which specializes in processing image information about an object in the receptive field^33^, the SC, a node in the visual pathway that contains neurons tuning to oculogyric cues^34^,^35^, provides a more reasonable assessment for comparing the utilization of vision among animals. And the IC is implicated in spatial hearing by encoding two-dimensional sound locations^14^. Therefore, different ratios of SC and IC volumes potentially indicate varied degrees of utilization of vision and hearing. Here, a much larger volume ratio of SC and IC (3:1) was found in Pteropodidae than the insectivorous bats (1:7), implied that Pteropodidae prefer vision, whereas insectivorous bats tend to use auditory signals to navigate.

The f-VEP has been widely applied in detecting animals’ visual perception thresholds^32^,^36^,^37^ and the f-VEP waveforms provided a more objective response of the central visual system than behavioral research when animals displayed behavioral learning deficits^38^,^39^. Furthermore, over the electroretinograms (ERGs), which only detect retinal photoreceptor capacity^40^, the f-VEP has advantages in detecting visual ability. The absolute visual thresholds (log cd/m^2^•s) of Pteropodidae (−6.30 and −6.37), that were even lower than those of rats (−4.99 for pigmented rats and −5.37 cd/m^2^•s for albino rats^32^,^41^), were lowest, following the Emballonuridae (−3.71) and insectivorous bats (−1.90). The results implied that Pteropodidae and Emballonuridae possessed better visual capacity than the insectivorous bat.

The individual component of f-VEP represents activation of the corresponding nucleus in the neural pathways during photic stimulation. The early cortical components are generated within thalamocortical radiations and appear as the most positive component with a latency of nearly 100 ms^42^,^43^. In *R. affinis* (the insectivorous bats), it was difficult to find a complete N-P-N structure and the first negative peak, which refers to pre-cortical neuronal activities in the visual pathway, was rarely observed either. By contrast, two peaks and one valley were always detected in Pteropodidae bats which may imply that more nuclei were supposed to participate in visual processing in Pteropodidae than the insectivorous bats.

Finally, to obtain the potential genetic basis of the divergent characteristics in the visual system, transcriptomes of five species of bats were sequenced, and a series of molecular evolution analyses was performed. First, positive selection was selected because the adaptive traits of organisms have evolved as a result of natural selection^44^. Among the genes analyzed, 112 positively selected genes (*P *≤ 0.05) were observed in Pteropodidae (branch a in Fig. 5), and after a FDR correction, 39 genes were retained (Table. S3). Some of these genes function directly in the visual pathway. For example, *PRPH2* encodes peripherin 2, which is essential for disk morphogenesis; defects in this gene are associated with retinal degeneration^45^. In addition, 160 positively selected genes (*P *≤ 0.05) were observed in Emballonuridae, another group of bats with good vision. After FDR correction, 81 of these PS genes remained (detailed in Fig. S4). These genes were enriched in the GO term mitochondrion (*P *= 0.016). Among these, 2 genes (*Rp1* and *CNGA1*) are directly related to vision. *Rp1* is required for the correct stacking of the outer segment disc and plays essential and synergistic roles that affect photosensitivity and the outer segment morphogenesis of rod cell photoreceptors^46^,^47^. *CNGA1* encodes a protein involved in phototransduction^48^,^49^. Natural selection of these genes, which function to capture photons and for phototransduction in the visual pathway, illustrates their importance for the evolution of better vision in these bats.

The convergent or parallel evolution of genes is an important mechanism of functional convergence^30^,^31^,^50^,^51^,^52^. Therefore, certain genes of the bat visual system were then evaluated to determine whether they had undergone convergent or parallel evolution. In total, 330 genes were detected to have signals of parallel evolution (Table S5). These genes were significantly enriched in terms of the sensory perception of light stimulus (GO: 0050953, *P *= 0.0110) and visual perception (GO: 0007601, *P *= 0.0110). Among these genes, *CRX,* which encodes the cone-rod homeobox protein, a photoreceptor-specific transcription factor essential for the differentiation of photoreceptor cells^53^, was also previously found to have undergone parallel evolution in Pteropodidae and Emballonuridae^31^. Similarly, *Rp1*, which is required for the normal morphogenesis of photoreceptor outer segments and may also play a role in rhodopsin transport to the outer segments^54^, displayed positive selection signals in Pteropodidae, indicating that multiple genes were likely involved in the visual evolution of bats.

Although amino acid changes may represent structural and functional variations in proteins and represent one typical strategy by which organisms adapt to the environment, another important mechanism of adaptation is represented by the organism’s gene expression profile. The expression pattern is commonly a strong indicator of protein demand and function^50^. Interestingly, significantly higher expression of genes related to camera-type eye development (GO: 0043010, *P *= 0.005) and eye development (GO: 0001654, *P *= 0.027) were found in Pteropodidae and Emballonuridae. In particular, a more highly expressed gene, *PRPH2,* which displayed a signal of positive selection in Pteropodidae, plays a critical role in the disk morphogenesis of the photoreceptor outer segment. Moreover, more than 40 different mutations in this gene have been associated with human retinopathies^55^. The higher expression and positive selection of *PRPH2* suggest that *PRPH2* may have played a role in the visual divergence of bats. In addition, the PCA showed that the expression pattern of Emballonuridae (*T. melanopogon*) was similar to that of Pteropodidae (*E. spelaea* and *C. sphinx*) and not that of the insectivorous bats (*H. armiger* and *M. laniger*). Furthermore, the extraordinarily high expression of the *RHO* gene in *T. melanopogon*, encoding a transmembrane protein that when photo excited, initiates the visual transduction cascade and is required for image-forming vision at low light intensity^56^,^57^, is a potential molecular mechanism underlying its normal vision compared with other insectivorous bats.

## Materials and Methods

The methods were carried out in accordance with the approved guidelines.

### Animals

The animals used here are detailed in Fig. S2. Before the f-VEP and MEMRI experiments, sample bats of all of the studied species were captured in the wild and housed in large cages (3 m x 3 m x 2.5 m) in a special animal room with a dark background luminance of 0.75 lx (AvaSpec-ULS2048LTEC, Avantes, Netherlands) and a controlled climate (temperature 22 ± 3 °C, humidity 80 ± 3%, filtered fresh air change rate of ~100% per hour). The Pteropodidae bats were provided with fruits and filtered tap water ad libitum; insectivorous bats were fed mealworms and filtered tap water artificially. Animals were allowed to acclimate to the facility for 5-6 days prior to the experiment.

Animal housing complied with the National Guidelines for the Care and Use of Animals and was approved by the National Animal Research Authority (P. R. China). All animal tests and experimental protocols were approved by the Ethics and Experimental Animal Committee of Kunming Institute of Zoology, Chinese Academy of Science, China (approval ID SWYX-20111010001).

### Manganese-enhanced magnetic resonance imaging (MEMRI)

#### MnCl_2_ administration

An isotonic solution of MnCl_2_•4H_2_O (Sigma-Aldrich, USA) was prepared in distilled water at 120 mM. For Pteropodidae, intravitreal injections with the Mn^2+^ solution at a dose of 2 μl/6 mm of their eye diameter were used to locate the visual pathway and the visual cortex. For Pteropodidae and the insectivorous bats, a dose of 3 ml/kg was used for intraperitoneal injections (i.p.) to display the structure of the whole brain, and SC and IC volume was obtained. After injections, the bats were allowed to recover from anesthesia and returned to their cages. Then, Pteropodidae were provided with fruits and water *ad libitum*, and the insectivorous bats were fed with mealworms and water. At 24 hours after the Mn^2+^ injection, the animals were placed in deep anesthesia under chloral hydrate (50 mg/0.1 kg, i.p.) for the MRI experiments.

#### MRI measurement

All MRI experiments were performed in a horizontal 7.0 T magnet equipped with a 6 mm rat head coil (Bruker, Germany). The head of the bat was placed in a head holder to minimize movement, and the body was laid on a water-based heated animal bed. Temperature and respiration were recorded using monitoring devices. A standard T1-weighted FLASH3D sequence was used to evaluate and identify regions enhanced by Mn^2+^ in the entire bat brain. MRI data acquisition specifications were as follows: field of view = 28.8 × 19.2 × 19.2 mm; image dimensions = 288 × 192 × 64 pixels; in plane resolution = 100 × 100 × 300 μm; Flip angle = 30; relaxation delay = 36 ms; echo time = 4.6 ms; number of average = 16, and total time ≈ 1.6 hours.

### Electrophysiological recording

For the f-VEP experiments, four species of bats, including Pteropodidae (*R. leschenaultii* and *C. sphinx*), insectivorous (*R. affinis*) and Emballonuridae (*T. melanopogon*) bats, were selected (details in Fig. S2). Two animals of *C. sphinx* (male, 26 g and 30 g in weight) and *R. leschenaultii* (male, 75 g and 88 g in weight) (Pteropodidae) were repeatedly measured in chronic recordings. Four animals each of *T. melanopogon* (22.5 g ± 2.60 g) and *R. affinis* (13.3 g ± 1.88 g) were used for acute measurement experiments because they did not survive long after surgery.

#### Electrode implantation surgery

Deep anesthesia (verified by absence of a tail-pinch reaction) was induced and maintained with sodium pentobarbital (3.4 mg/0.1 kg, i.p.). For f-VEP recording, epidural stainless steel screw electrodes^58^,^59^ (0.9 mm in diameter, tip area of 0. mm^2^) were stereotaxically implanted after the skin and muscle were resected. The active electrodes were placed over the bilateral visual cortex (V1) (relative location to the bone marker according to the results of MEMRI, Fig. 4), and the reference and ground electrodes were placed over the frontal cortical area (2 mm lateral and 2 mm anterior to the bregma, ipsilateral to the active electrode). The entire implant, including additional screws, was fixed to the skull with glass ionomer dental cement.

#### Evoked potential procedure and data analyses

The test room was illuminated with a dim red safelight. Before f-VEP recording, the bats were restrained in a tubular restraint device to fix their heads and wing flaps and then acclimated in absolute darkness (less than 0.25 lx luminescence) for 20 minutes. Recordings of evoked potentials were taken from bilateral cortices for each experiment; simulation was unilateral, and the other eye was covered with an opaque black blanket.

Photic stimuli were generated using Psychophysics Toolbox Version 3 (PTB-3, MATLAB, R2009b) and presented on a 21 inch computer display (1600 × 900 @ 120 Hz, LCD, Samsung, Korea) suitably linearized by gamma correction. The computer screen was placed 16.78 cm in front of the bat’s eye to obtain consistent light input. All bats were stimulated at 12 levels (−0.95, −1.49, −1.90, −2.45, −2.85, −3.71, −4.77, −6.20, −6.30, −6.37, −6.43, and −6.51  log cd/m^2^•s) of photic intensity. The background noise of the testing room was 45 dB near the animal’s ear, and no obvious changes in sound intensity occurred when the display flashed (Digital Sound Level Meter AR814, SMART SENSOR, Hong Kong).

For each VEP experiment, two trains of 180 flash stimuli at a 100 ms duration were generated. The baseline section used the same stimuli but with the bat draped under an opaque black blanket. Evoking stimuli were presented with an ISI of 1 to 3 s (0.3–1 Hz, random). Evoked potentials were amplified with SYMTOP preamplifiers (Symtop, UEA-41FZ, CHN) with a 1000 Hz sample rate and low and high cutoff frequencies of 0.1 and 150 Hz, respectively. Absolute visual threshold detection was conducted from 18:00 to 21:00, which is the normal active period of the animals, to ensure a relatively stable physiological state.

Amplified waveforms were averaged in EEGLAB 6.0 (MATLAB toolbox, EEGLAB 6.0) by filtering the data with a digital finite impulse response filter (band-pass with 1–50 Hz); 180 epochs were averaged with a 1000 ms epoch and a 100 ms pre-epoch per f-VEP. Peak heights were measured relative to 0 amplitude; latencies were measured from flash onset to the respective peaks. Rather than focusing on a single peak, an N-P-N structure was used to extrapolate the absolute visual thresholds of the bats. The baseline was a mean value of the ratio of the highest amplitude of waveforms generated without stimulation to evoke the P1 (utmost peak) amplitude at the highest photic intensity level across 4 bats.

### Whole-transcriptome shotgun sequencing (RNA-seq)

#### Sample collection and library preparation

For sampling, bats were anesthetized using an intraperitoneal injection of sodium pentobarbital at a dosage of 100 mg per kg body weight, and the eyes were sampled immediately, frozen in liquid nitrogen and preserved at −80 °C. When extracting RNA, TRIzol (Invitrogen Corp., Carlsbad, CA) was used for lysis, and the RNeasy Mini Kit (Qiagen, Chatsworth, CA) was used for total RNA purification, and DNA was digested with DNase before library construction. RNA from the eyes of 2 and 3 individuals of *M. laniger* and *H. armiger*, respectively, was pooled together before library construction because of their small eye size.

The cDNA libraries were constructed according to the protocol provided by Illumina (kit RS-930-1001, #1004898 Rev. D). The library was validated and quantified using the Qubit 1.0 (Life Technologies Inc., India) and the Agilent 2100 Bioanalyzer (Agilent, Inc.). The libraries from *C. sphinx*, *H. armiger,* and *M. laniger* were sequenced in the HiSeq 2000 (Illumina Inc.) using the 2 × 101 cycle setting. Libraries from *E. spelaea* and *T. melanopogon* were sequenced in the Illumina Genome Analyzer IIx (GA IIx, Illumina Inc.) under the 2 × 87 cycle setting.

#### De novo assembly, open reading frame (ORF) prediction and annotation

Cutadapt software (http://code.google.com/p/cutadapt/) was used to filter out the adapters, and Btrim software (http://graphics.med.yale.edu/trim/) was used to trim the low-quality bases (quality score lower than 20). Trinity (version 2012-04-27)^60^ was used to assemble more full-length transcripts with lengths longer than 200 bp. CD-HIT-EST^61^ (version 4.5.4) was used to remove redundancy with default parameters. The ‘-getorf’ function in the EMBOSS (http://emboss.open-bio.org/wiki/Appdocs) package was used to extract all possible ORFs with ‘-find 2’ and ‘-find 3’. Next, an in-house script was used to find the longest ORF. Then, the longest ORF (regarded as the predicted coding sequence) was translated into amino acids with transeq in the EMBOSS package and annotated with the little brown bat protein sequences (ENSEMBL, version 70) using a reciprocal best-hit BLASTp search with an e-value of 1e-6.

#### Multiple alignments

Genes of the five sequenced transcriptomes annotated with the same little brown bat gene were considered one-to-one orthologs. Then, the ENSEMBL ortholog one2one gene database was used to search for orthologs between the little brown bat and dog, which was used as the out-group in the subsequent analysis. Multiple alignments were conducted using the program PRANK (-F -codon -noxml -notree -nopost, http://www.ebi.ac.uk/goldman-srv/prank/prank/) and selecting the longest transcript from each species. Finally, several trimming strategies were performed^44^; i.e., all gaps and “N” bases from the alignments were discarded from the respective codon units, a 15-bp sliding window was moved along the alignment, and alignment regions whose lowest similarity of an alignment pair in the 6 species was <7/15 were discarded. Alignments longer than 100 nt were retained.

#### Selective pressure analyses and parallel/convergent evolution

The CODEML algorithm from the PAML 4 package^62^ was used for ancestor sequence reconstruction and testing for selection with branch-site (the null model: model = 2, NSsites = 2, fix_omega = 0; the alternative model: model = 2, NSsites = 2, fix_omega = 1) model and test2 using the accepted phylogenetic relationships within Chiroptera ((((*C. sphinx*, *E. spelaea*), *H. armiger*), (*T. melanopogon*, *M. laniger*)), *C. familiaris*)^63^, with dog serving as the out-group. The reconstructed amino acid sites with posterior probabilities more than 90% and the numbers of convergent/parallel amino acid replacements were counted for each pair of branches using a custom script. Only extremely well-conserved sequences, with only two amino acid states in all the species, were considered parallel sites and saved for further analysis.

#### Expression profiling and PCA analysis

In these analyses, all reads from the five species of bats were merged to construct another assembly using SOAP *de novo*-trans (version 1.0 http://soap.genomics.org.cn/ SOAPdenovo-Trans.html) with a k-mer range from 25–75 and a step value of 5. Then, the contigs of the five species of bats assembled in Trinity were mixed, and the redundancy was removed. First, the *de novo* non-redundant transcripts were annotated using BLASTx alignment with the little brown bat (ENSEMBL 70) as the database and an e-value of 1e-6. Either the contigs blasted to only one gene, which was passed on for further downstream analysis, or a ratio less than 0.7 between the BLAST score of the ‘second-best-hit-gene’ to the BLAST score of the ‘first-best-hit-gene’ was used to reduce the influence of gene families passed on to the downstream analysis.

RSEM^64^ was then used to summarize the gene expression levels of the studied species with default parameter. Here, only genes with a count ≥1 from all species were summarized and passed on as the input of Edger (http://www.bioconductor.org/, adjustment method: Benjamini and Hochberg’s approach for controlling the false discovery rate (FDR)).

Last, a PCA^65^ using an R script (available at http://stat.ethz.ch/R-manual/R-patched/library/stats/html/princomp.html) was conducted to summarize the features of the expression profiles in each transcriptome.

#### Functional annotation clustering

The database for Annotation, Visualization, and Integrated Discovery (DAVID)^66^ was used to cluster the genes of interest.

## Additional Information

**How to cite this article**: Liu, H.-Q. *et al.* Divergence of dim-light vision among bats (order: Chiroptera) as estimated by molecular and electrophysiological methods. *Sci. Rep.* **5**, 11531; doi: 10.1038/srep11531 (2015).

## Supplementary Material

Supplementary Information

## Figures and Tables

**Table 1:** Latencies of N-P-N wave components in 4 bat species. Mean ± SD, n = 12 (n = 5 in *R. affinis*).

**Latency (ms)**				
	***R. leschenaultii***	***C. sphinx***	***T. melanopogon***	***R. affinis***
N1	42.7 ± 10.9	35.8 ± 5.4	59.2 ± 10.7	–
P1	102.3 ± 13.49	89.0 ± 11.1	105.4 ± 8.8	117.8 ± 8.0
N2 (N1 for *R. affinis*)	175.9 ± 24.5	140.6 ± 16.2	141.5 ± 10.0	196.0 ± 11.8
