# Supplementary material for: Divergence of dim-light vision among bats (order: Chiroptera) as estimated by molecular and electrophysiological methods
Source: Sci Rep. 2015 Jun 23;5:11531. doi: 10.1038/srep11531 (PMC5155579; doi:10.1038/srep11531)
Supplement: Supplementary Information [file srep11531-s1.doc]

**Divergence of dim-light vision among bats (order: Chiroptera) as estimated by molecular and electrophysiological methods**

He-Qun Liu^1,6,7,#^, Jing-Kuan Wei^2,6,7,#^, Bo Li^4,7,#^, Ming-Shan Wang^1,6,7^, Rui-Qi Wu^4,7^, Joshua D. Rizak.^2,7^, Li Zhong^5^, Lu Wang^5^, Fu-Qiang Xu^4^, Yong-Yi Shen^1,3*^, Xin-Tian Hu^2*^ and Ya-Ping Zhang^1,5,6*^

1. State Key Laboratory of Genetic Resources and Evolution, Yunnan Laboratory of Molecular Biology of Domestic Animals, Kunming Institute of Zoology, Chinese Academy of Sciences, Kunming, 650223, China;
2. Key Laboratory of Animal Models and Human Disease Mechanisms, Kunming Institute of Zoology, Chinese Academy of Sciences, Kunming, 650223, China;
3. Joint Influenza Research Centre (SUMC/HKU), Shantou University Medical College, Shantou, 515041, China;
4. State Key Laboratory of Magnetic Resonance and Atomic and Molecular Physics, and Key Laboratory of Magnetic Resonance in Biological Systems, Wuhan Institute of Physics and Mathematics, Chinese Academy of Sciences, Wuhan, 430071, China;
5. Laboratory for Conservation and Utilization of Bio-resource, Yunnan University, Kunming, 650091, China;
6. Kunming College of Life Science, University of the Chinese Academy of Sciences, Kunming, 650204, China;
7. University of the Chinese Academy of Sciences, Beijing, China.

#These authors contributed equally to this work.

*To whom correspondence should be addressed:

Dr. Ya-Ping Zhang,

Email: zhangyp@mail.kiz.ac.cn

Dr. Xin-Tian Hu,

Email: xthu@mail.kiz.ac.cn

Dr. Yong-Yi Shen,

Email: shenyy@mail.kiz.ac.cn


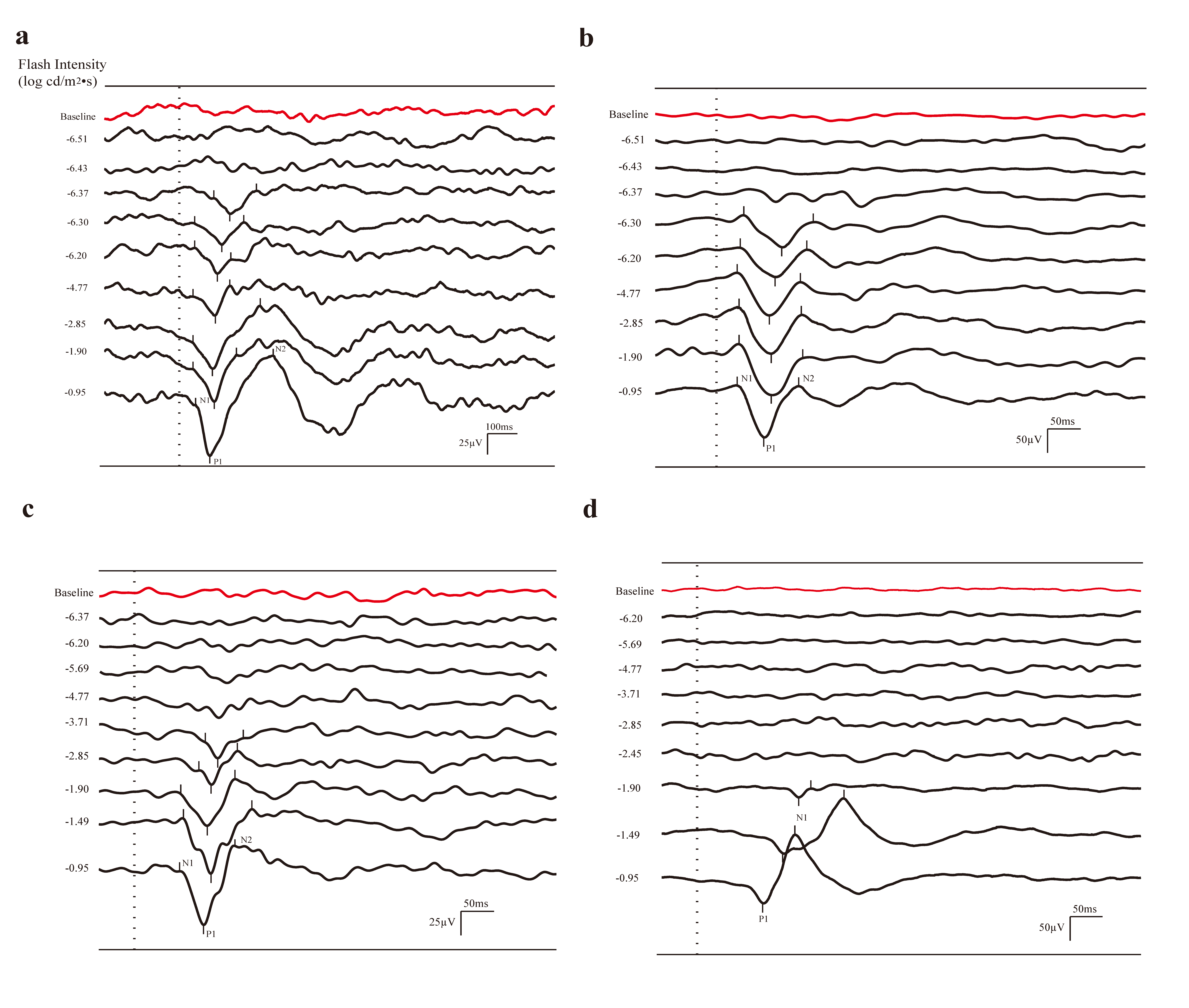
**Figure S1. Representative f-VEP waveforms recorded in each bat species at different light intensities.** Vertical dot lines represent onset of the stimulus. (a) *R. leschenaultii*; (b) *C. sphinx*; (c) *T. melanopogon*; (d) *R. affinis*

**Figure S2. List of bats and methods used in this study** (The photographs of *R. affinis, R. leschenaultii, C. sphinx* and *M. laniger* were taken by H.Q. L., and photographs of *H.amiger*, *E. spelea* and *T. melanopogon* were obtained from
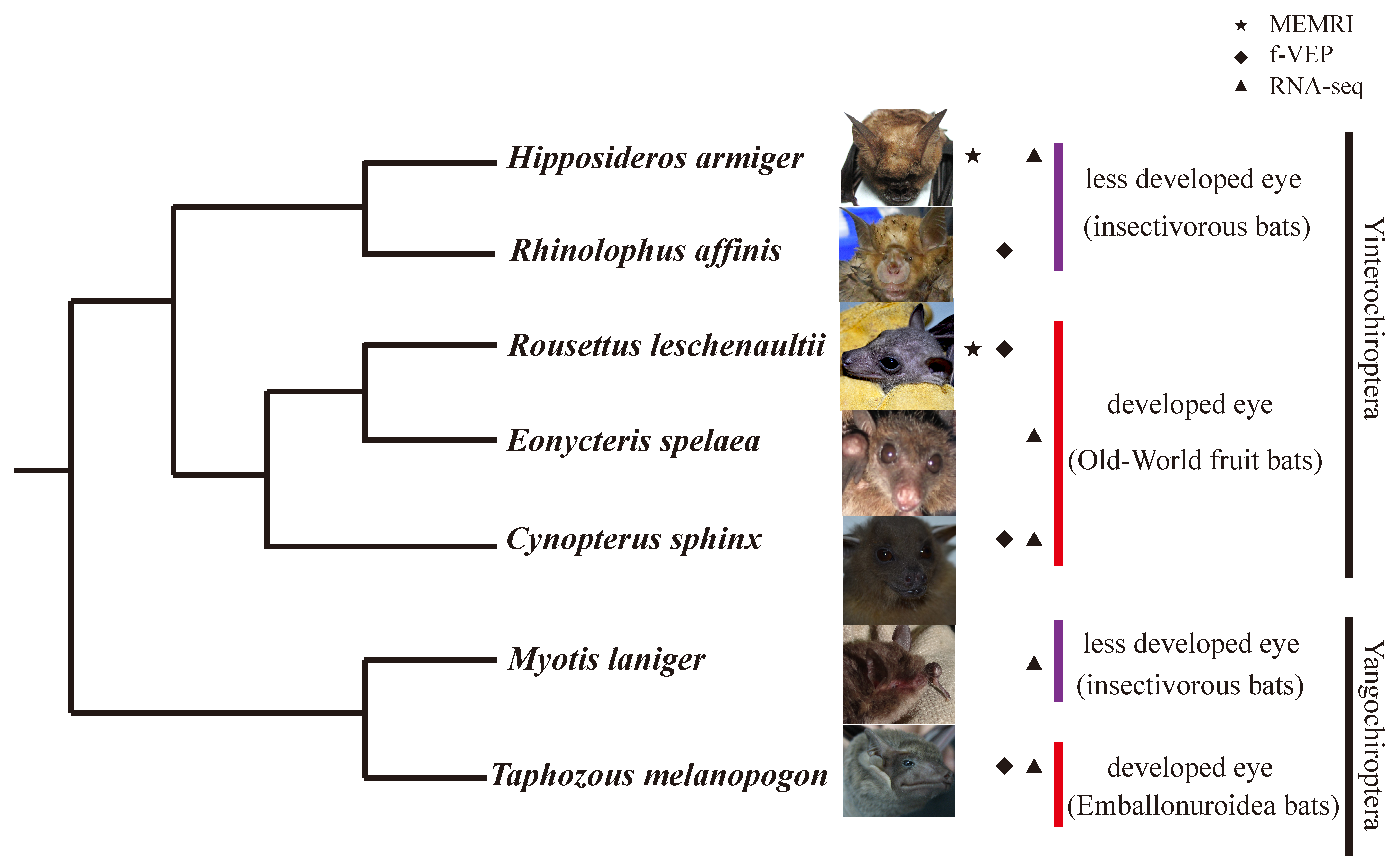
Xu-Dong Zhao.)

**Table S1. Detailed sequencing information of each sample**

| samples | terminal | Raw reads base(G) | Raw reads number | Reads btrimed base (G) | Reads btrimed number |
| --- | --- | --- | --- | --- | --- |
| *Taphozous melanopogon* | read1 | 3.704193320 | 38456715 | 2.013806188 | 29768597 |
| *Taphozous melanopogon* | read2 | 3.704193320 | 38456715 | 1.976439436 | 29768597 |
| *Cynopterus sphinx* | read1 | 3.120869922 | 32724693 | 2.509055057 | 27577449 |
| *Cynopterus sphinx* | read2 | 3.120869922 | 32724693 | 2.448928838 | 27577449 |
| *Myotis laniger* | read1 | 2.770211887 | 29047777 | 2.332023491 | 25783869 |
| *Myotis laniger* | read2 | 2.770211887 | 29047777 | 2.278411861 | 25783869 |
| *Eonycteris spelaea* | read1 | 3.942191317 | 47513646 | 3.299464026 | 44347976 |
| *Eonycteris spelaea* | read2 | 3.942191317 | 47513646 | 3.406834527 | 44347976 |
| *Hipposideros armiger* | read1 | 3.266211815 | 32931455 | 2.753397810 | 30004511 |
| *Hipposideros armiger* | read2 | 3.266211815 | 32931455 | 2.732360989 | 30004511 |
| total | Pair-end | 33.61 |  | 25.75 |  |

**Table S2. Detailed assembly and annotation information of each transcriptome**

| Species | N50(bp) | N90(bp) | longest contig(bp) | one2one genes |
| --- | --- | --- | --- | --- |
| *Taphozous melanopogon* | 2253 | 309 | 18460 | 11389 |
| *Cynopterus sphinx* | 1100 | 299 | 19414 | 10276 |
| *Myotis laniger* | 1256 | 278 | 17727 | 10755 |
| *Eonycteris spelaea* | 2380 | 306 | 25539 | 11799 |
| *Hipposideros armiger* | 1643 | 320 | 23142 | 11644 |
| average | 1726 | 302 | 20856 | 11172 |

**Table S3. Genes that show signals of positive selection as tested by the branch-site model with branch a as the foreground**

| gene | p values | FDR |
| --- | --- | --- |
| ENSCAFG00000000012 | 3.08E-15 | 5.38E-13 |
| ENSCAFG00000000043 | 0.045464 | 0.075059 |
| ENSCAFG00000000212 | 0.087743 | 0.094202 |
| ENSCAFG00000000406 | 0.010089 | 0.045272 |
| ENSCAFG00000000601 | 0.025861 | 0.066554 |
| ENSCAFG00000000607 | 0.002871 | 0.026443 |
| ENSCAFG00000000671 | 0.091294 | 0.095667 |
| ENSCAFG00000000830 | 0.025919 | 0.065737 |
| ENSCAFG00000000848 | 0.022474 | 0.06555 |
| ENSCAFG00000000867 | 0.013793 | 0.053641 |
| ENSCAFG00000000954 | 0.01006 | 0.046328 |
| ENSCAFG00000001047 | 0.015876 | 0.056699 |
| ENSCAFG00000001293 | 0.034936 | 0.065739 |
| ENSCAFG00000001330 | 0.073178 | 0.090823 |
| ENSCAFG00000001586 | 0.022082 | 0.065498 |
| ENSCAFG00000001586 | 0.022082 | 0.066628 |
| ENSCAFG00000001598 | 0.031684 | 0.071085 |
| ENSCAFG00000001640 | 0.001025 | 0.01793 |
| ENSCAFG00000001722 | 0.027122 | 0.066851 |
| ENSCAFG00000001767 | 0.036173 | 0.067343 |
| ENSCAFG00000002188 | 0.002441 | 0.025127 |
| ENSCAFG00000002226 | 0.085383 | 0.093388 |
| ENSCAFG00000002711 | 0.000914 | 0.017766 |
| ENSCAFG00000002821 | 0.046071 | 0.075349 |
| ENSCAFG00000003202 | 0.0847 | 0.094411 |
| ENSCAFG00000003301 | 0.001266 | 0.017036 |
| ENSCAFG00000003498 | 0.037235 | 0.068591 |
| ENSCAFG00000003617 | 0.021185 | 0.068654 |
| ENSCAFG00000003663 | 0.007959 | 0.042204 |
| ENSCAFG00000003688 | 0.08568 | 0.093131 |
| ENSCAFG00000003723 | 0.037557 | 0.068464 |
| ENSCAFG00000003969 | 0.07469 | 0.090143 |
| ENSCAFG00000004011 | 0.08513 | 0.093697 |
| ENSCAFG00000004093 | 0.021566 | 0.067393 |
| ENSCAFG00000004223 | 0.084292 | 0.094559 |
| ENSCAFG00000004332 | 0.047233 | 0.076534 |
| ENSCAFG00000004496 | 0.062382 | 0.083975 |
| ENSCAFG00000004501 | 0.01497 | 0.055739 |
| ENSCAFG00000004516 | 0.034522 | 0.067126 |
| ENSCAFG00000004646 | 0.034586 | 0.066512 |
| ENSCAFG00000004906 | 0.025178 | 0.067787 |
| ENSCAFG00000005034 | 0.069484 | 0.088114 |
| ENSCAFG00000005039 | 0.074637 | 0.091339 |
| ENSCAFG00000005282 | 0.087964 | 0.093295 |
| ENSCAFG00000005809 | 0.074648 | 0.090718 |
| ENSCAFG00000005918 | 0.024588 | 0.069403 |
| ENSCAFG00000005919 | 0.004514 | 0.032914 |
| ENSCAFG00000005948 | 0.006191 | 0.038692 |
| ENSCAFG00000005993 | 0.006471 | 0.039049 |
| ENSCAFG00000006312 | 0.012256 | 0.051067 |
| ENSCAFG00000006591 | 0.048137 | 0.076582 |
| ENSCAFG00000006702 | 0.034299 | 0.068209 |
| ENSCAFG00000006938 | 0.053617 | 0.078191 |
| ENSCAFG00000007007 | 0.053525 | 0.079381 |
| ENSCAFG00000007218 | 0.034759 | 0.066117 |
| ENSCAFG00000007261 | 0.000896 | 0.022388 |
| ENSCAFG00000007498 | 0.080283 | 0.093663 |
| ENSCAFG00000007669 | 0.095605 | 0.099 |
| ENSCAFG00000007670 | 0.033241 | 0.069252 |
| ENSCAFG00000007731 | 0.012788 | 0.052044 |
| ENSCAFG00000007745 | 0.068478 | 0.088768 |
| ENSCAFG00000007749 | 0.029303 | 0.070247 |
| ENSCAFG00000007945 | 0.053615 | 0.078846 |
| ENSCAFG00000008087 | 0.058341 | 0.081677 |
| ENSCAFG00000008143 | 0.01602 | 0.054971 |
| ENSCAFG00000008249 | 0.06533 | 0.086611 |
| ENSCAFG00000008349 | 0.060252 | 0.082376 |
| ENSCAFG00000008438 | 0.001502 | 0.018772 |
| ENSCAFG00000008673 | 0.026877 | 0.067192 |
| ENSCAFG00000009076 | 0.078707 | 0.092441 |
| ENSCAFG00000009143 | 0.02483 | 0.068972 |
| ENSCAFG00000009409 | 0.048646 | 0.076695 |
| ENSCAFG00000009617 | 0.038142 | 0.068812 |
| ENSCAFG00000009651 | 0.04156 | 0.07201 |
| ENSCAFG00000009822 | 0.009973 | 0.047168 |
| ENSCAFG00000009846 | 0.032135 | 0.070295 |
| ENSCAFG00000010131 | 0.013693 | 0.054461 |
| ENSCAFG00000010145 | 0.027323 | 0.066409 |
| ENSCAFG00000010161 | 0.054506 | 0.07883 |
| ENSCAFG00000010296 | 0.030731 | 0.069843 |
| ENSCAFG00000010449 | 0.041659 | 0.071473 |
| ENSCAFG00000010730 | 0.038581 | 0.068894 |
| ENSCAFG00000010759 | 0.044575 | 0.075007 |
| ENSCAFG00000010857 | 0.066823 | 0.087924 |
| ENSCAFG00000010928 | 0.098098 | 0.099232 |
| ENSCAFG00000010994 | 0.041396 | 0.072443 |
| ENSCAFG00000011216 | 0.087829 | 0.09372 |
| ENSCAFG00000011303 | 0.099373 | 0.099944 |
| ENSCAFG00000011671 | 0.007951 | 0.043481 |
| ENSCAFG00000011770 | 0.064658 | 0.086375 |
| ENSCAFG00000011948 | 0.002234 | 0.026061 |
| ENSCAFG00000012103 | 0.090051 | 0.094934 |
| ENSCAFG00000012224 | 0.029418 | 0.068643 |
| ENSCAFG00000012638 | 0.03882 | 0.068621 |
| ENSCAFG00000012666 | 0.003978 | 0.031645 |
| ENSCAFG00000012998 | 0.005704 | 0.036971 |
| ENSCAFG00000012999 | 0.044897 | 0.074828 |
| ENSCAFG00000013075 | 0.034464 | 0.067765 |
| ENSCAFG00000013076 | 0.099463 | 0.099463 |
| ENSCAFG00000013089 | 0.009002 | 0.045009 |
| ENSCAFG00000013205 | 0.029317 | 0.069332 |
| ENSCAFG00000013390 | 0.084767 | 0.093887 |
| ENSCAFG00000013817 | 0.000102 | 0.004442 |
| ENSCAFG00000013878 | 0.000565 | 0.016485 |
| ENSCAFG00000014220 | 0.096544 | 0.098802 |
| ENSCAFG00000014222 | 0.050149 | 0.077665 |
| ENSCAFG00000014332 | 0.074988 | 0.089883 |
| ENSCAFG00000014373 | 0.032407 | 0.069161 |
| ENSCAFG00000014533 | 0.009271 | 0.045065 |
| ENSCAFG00000014584 | 0.0527 | 0.079505 |
| ENSCAFG00000014610 | 0.011457 | 0.050124 |
| ENSCAFG00000014624 | 0.006879 | 0.040129 |
| ENSCAFG00000014749 | 0.016172 | 0.054425 |
| ENSCAFG00000014755 | 0.01197 | 0.05109 |
| ENSCAFG00000014821 | 0.098056 | 0.099766 |
| ENSCAFG00000014939 | 0.080919 | 0.093164 |
| ENSCAFG00000014984 | 0.001155 | 0.016846 |
| ENSCAFG00000015143 | 0.004815 | 0.033707 |
| ENSCAFG00000015383 | 0.078547 | 0.092876 |
| ENSCAFG00000015398 | 0.03232 | 0.069828 |
| ENSCAFG00000015537 | 0.087148 | 0.094141 |
| ENSCAFG00000015576 | 0.043967 | 0.074702 |
| ENSCAFG00000015659 | 0.052419 | 0.079767 |
| ENSCAFG00000015710 | 0.059073 | 0.0814 |
| ENSCAFG00000015805 | 0.057138 | 0.081293 |
| ENSCAFG00000015825 | 0.033984 | 0.069154 |
| ENSCAFG00000015832 | 0.068858 | 0.087958 |
| ENSCAFG00000015978 | 0.03401 | 0.06841 |
| ENSCAFG00000016027 | 0.001113 | 0.017713 |
| ENSCAFG00000016168 | 0.081181 | 0.092854 |
| ENSCAFG00000016185 | 0.030204 | 0.069549 |
| ENSCAFG00000016286 | 0.004337 | 0.032996 |
| ENSCAFG00000016954 | 0.000444 | 0.015538 |
| ENSCAFG00000016962 | 0.025763 | 0.067292 |
| ENSCAFG00000017037 | 0.023241 | 0.066676 |
| ENSCAFG00000017077 | 0.052407 | 0.08045 |
| ENSCAFG00000017142 | 0.094048 | 0.097966 |
| ENSCAFG00000017144 | 2.77E-07 | 2.42E-05 |
| ENSCAFG00000017640 | 0.033292 | 0.068543 |
| ENSCAFG00000017715 | 0.047665 | 0.076526 |
| ENSCAFG00000017784 | 0.032755 | 0.069063 |
| ENSCAFG00000017884 | 0.019347 | 0.063883 |
| ENSCAFG00000018024 | 0.082612 | 0.093877 |
| ENSCAFG00000018064 | 4.84E-05 | 0.002822 |
| ENSCAFG00000018285 | 0.058836 | 0.081716 |
| ENSCAFG00000018641 | 0.068761 | 0.08848 |
| ENSCAFG00000018647 | 0.080667 | 0.093488 |
| ENSCAFG00000018720 | 0.074369 | 0.091651 |
| ENSCAFG00000018933 | 0.021548 | 0.06856 |
| ENSCAFG00000019019 | 0.005159 | 0.034724 |
| ENSCAFG00000019140 | 0.060556 | 0.082149 |
| ENSCAFG00000019346 | 0.002882 | 0.025221 |
| ENSCAFG00000019490 | 0.084006 | 0.094846 |
| ENSCAFG00000019677 | 0.067921 | 0.088702 |
| ENSCAFG00000019687 | 0.05507 | 0.078993 |
| ENSCAFG00000019865 | 0.049755 | 0.077743 |
| ENSCAFG00000019963 | 0.015878 | 0.055573 |
| ENSCAFG00000020014 | 0.031878 | 0.070615 |
| ENSCAFG00000020157 | 0.025639 | 0.067982 |
| ENSCAFG00000023230 | 0.002435 | 0.026636 |
| ENSCAFG00000023549 | 0.052754 | 0.078906 |
| ENSCAFG00000023645 | 0.013881 | 0.052809 |
| ENSCAFG00000023963 | 0.00258 | 0.025086 |
| ENSCAFG00000024432 | 0.015798 | 0.057595 |
| ENSCAFG00000024785 | 0.000905 | 0.019787 |
| ENSCAFG00000025565 | 0.021571 | 0.066225 |
| ENSCAFG00000029750 | 0.06964 | 0.087677 |
| ENSCAFG00000029971 | 0.008373 | 0.043096 |
| ENSCAFG00000030193 | 0.058013 | 0.081873 |
| ENSCAFG00000030331 | 0.003382 | 0.02818 |
| ENSCAFG00000031506 | 0.072713 | 0.090891 |
| ENSCAFG00000031608 | 0.007104 | 0.040105 |
| ENSCAFG00000031675 | 0.07723 | 0.09194 |
| ENSCAFG00000031897 | 0.095911 | 0.098732 |
| ENSCAFG00000032102 | 0.024862 | 0.067982 |

**Table S4.** Genes that show signals of positive selection as tested by the branch-site model with branch b as the foreground

| gene | p values | FDR |
| --- | --- | --- |
| ENSCAFG00000000260 | 0.003404 | 0.028137 |
| ENSCAFG00000000348 | 0.040011 | 0.07405 |
| ENSCAFG00000000373 | 8.32E-07 | 6.88E-05 |
| ENSCAFG00000000463 | 0.064584 | 0.087523 |
| ENSCAFG00000000533 | 0.01068 | 0.04342 |
| ENSCAFG00000000601 | 0.001418 | 0.018511 |
| ENSCAFG00000000664 | 0.018128 | 0.051676 |
| ENSCAFG00000000701 | 0.00809 | 0.038581 |
| ENSCAFG00000000823 | 0.001202 | 0.01863 |
| ENSCAFG00000001047 | 0.015876 | 0.049215 |
| ENSCAFG00000001069 | 0.017986 | 0.051868 |
| ENSCAFG00000001075 | 0.092102 | 0.099744 |
| ENSCAFG00000001086 | 0.080347 | 0.093112 |
| ENSCAFG00000001141 | 0.071724 | 0.090292 |
| ENSCAFG00000001416 | 0.024343 | 0.05642 |
| ENSCAFG00000001900 | 0.087309 | 0.096664 |
| ENSCAFG00000001917 | 0.005417 | 0.033587 |
| ENSCAFG00000002079 | 0.090367 | 0.099605 |
| ENSCAFG00000002090 | 0.001236 | 0.018032 |
| ENSCAFG00000002098 | 0.035537 | 0.067276 |
| ENSCAFG00000002220 | 0.068797 | 0.089328 |
| ENSCAFG00000002313 | 0.002012 | 0.020788 |
| ENSCAFG00000002492 | 0.086864 | 0.096602 |
| ENSCAFG00000002697 | 0.023405 | 0.058045 |
| ENSCAFG00000002821 | 0.046071 | 0.074191 |
| ENSCAFG00000002940 | 0.006718 | 0.037865 |
| ENSCAFG00000003077 | 0.01235 | 0.047121 |
| ENSCAFG00000003094 | 0.054439 | 0.080844 |
| ENSCAFG00000003192 | 0.002708 | 0.023159 |
| ENSCAFG00000003380 | 0.064157 | 0.088395 |
| ENSCAFG00000003498 | 0.037235 | 0.06943 |
| ENSCAFG00000003577 | 0.01722 | 0.050839 |
| ENSCAFG00000003583 | 0.007261 | 0.036751 |
| ENSCAFG00000003803 | 0.00842 | 0.03867 |
| ENSCAFG00000003821 | 0.045537 | 0.075288 |
| ENSCAFG00000003867 | 0.013821 | 0.048277 |
| ENSCAFG00000004006 | 0.005042 | 0.032907 |
| ENSCAFG00000004351 | 0.079685 | 0.093216 |
| ENSCAFG00000004407 | 0.084178 | 0.095762 |
| ENSCAFG00000004652 | 0.052943 | 0.080552 |
| ENSCAFG00000004862 | 0.09583 | 0.101132 |
| ENSCAFG00000004926 | 0.023558 | 0.057277 |
| ENSCAFG00000004959 | 0.050297 | 0.077477 |
| ENSCAFG00000005214 | 0.015626 | 0.049683 |
| ENSCAFG00000005378 | 0.090968 | 0.099384 |
| ENSCAFG00000005387 | 0.024249 | 0.056733 |
| ENSCAFG00000005469 | 0.066867 | 0.088679 |
| ENSCAFG00000005564 | 0.071713 | 0.090739 |
| ENSCAFG00000005919 | 0.004514 | 0.031096 |
| ENSCAFG00000005936 | 0.063873 | 0.088495 |
| ENSCAFG00000006160 | 0.000595 | 0.014751 |
| ENSCAFG00000006222 | 0.010273 | 0.04246 |
| ENSCAFG00000006269 | 0.027026 | 0.060382 |
| ENSCAFG00000006502 | 0.00015 | 0.006185 |
| ENSCAFG00000006521 | 0.017893 | 0.052206 |
| ENSCAFG00000006847 | 0.076206 | 0.09219 |
| ENSCAFG00000006960 | 0.096656 | 0.100718 |
| ENSCAFG00000006979 | 0.079142 | 0.093463 |
| ENSCAFG00000006995 | 4.93E-05 | 0.002447 |
| ENSCAFG00000007288 | 0.001966 | 0.021203 |
| ENSCAFG00000007470 | 0.064739 | 0.087256 |
| ENSCAFG00000007580 | 0.022152 | 0.056637 |
| ENSCAFG00000007588 | 0.000214 | 0.007568 |
| ENSCAFG00000007629 | 0.02499 | 0.057384 |
| ENSCAFG00000007669 | 0.002448 | 0.021683 |
| ENSCAFG00000007948 | 0.071114 | 0.090442 |
| ENSCAFG00000008037 | 0.041361 | 0.075423 |
| ENSCAFG00000008060 | 0.016595 | 0.050191 |
| ENSCAFG00000008292 | 0.017093 | 0.051073 |
| ENSCAFG00000008331 | 0.041485 | 0.075097 |
| ENSCAFG00000008349 | 0.055818 | 0.081911 |
| ENSCAFG00000008351 | 0.060999 | 0.085954 |
| ENSCAFG00000008352 | 0.000312 | 0.008598 |
| ENSCAFG00000008424 | 0.096664 | 0.100304 |
| ENSCAFG00000008478 | 0.05431 | 0.081139 |
| ENSCAFG00000008892 | 0.012836 | 0.046814 |
| ENSCAFG00000008901 | 0.013678 | 0.04846 |
| ENSCAFG00000008923 | 0.014984 | 0.049546 |
| ENSCAFG00000009069 | 0.097478 | 0.099895 |
| ENSCAFG00000009174 | 0.007472 | 0.037062 |
| ENSCAFG00000009320 | 0.075314 | 0.091558 |
| ENSCAFG00000009334 | 0.018337 | 0.050528 |
| ENSCAFG00000009409 | 0.01362 | 0.048953 |
| ENSCAFG00000009421 | 0.006819 | 0.037579 |
| ENSCAFG00000009577 | 0.045821 | 0.07476 |
| ENSCAFG00000009651 | 0.018472 | 0.050341 |
| ENSCAFG00000009797 | 0.001448 | 0.017952 |
| ENSCAFG00000009819 | 0.094466 | 0.100548 |
| ENSCAFG00000009846 | 0.032135 | 0.065324 |
| ENSCAFG00000010170 | 0.073242 | 0.091277 |
| ENSCAFG00000010172 | 0.015736 | 0.0494 |
| ENSCAFG00000010307 | 0.023808 | 0.057324 |
| ENSCAFG00000010366 | 0.013969 | 0.048115 |
| ENSCAFG00000010449 | 0.041659 | 0.074326 |
| ENSCAFG00000010630 | 0.000743 | 0.01535 |
| ENSCAFG00000010662 | 0.074693 | 0.091251 |
| ENSCAFG00000010674 | 0.029416 | 0.062352 |
| ENSCAFG00000010687 | 0.003757 | 0.030052 |
| ENSCAFG00000010928 | 0.098098 | 0.099706 |
| ENSCAFG00000010995 | 0.015967 | 0.048887 |
| ENSCAFG00000011216 | 0.032686 | 0.063828 |
| ENSCAFG00000011247 | 0.023954 | 0.056577 |
| ENSCAFG00000011303 | 0.099373 | 0.099373 |
| ENSCAFG00000011740 | 0.028546 | 0.062099 |
| ENSCAFG00000011802 | 0.07644 | 0.091581 |
| ENSCAFG00000011865 | 0.068207 | 0.089499 |
| ENSCAFG00000011896 | 0.023459 | 0.057602 |
| ENSCAFG00000011924 | 0.036195 | 0.068002 |
| ENSCAFG00000011930 | 0.003923 | 0.030401 |
| ENSCAFG00000011940 | 0.061712 | 0.086467 |
| ENSCAFG00000011957 | 0.029309 | 0.062661 |
| ENSCAFG00000012140 | 0.021568 | 0.056304 |
| ENSCAFG00000012152 | 0.028724 | 0.061945 |
| ENSCAFG00000012274 | 0.010835 | 0.042651 |
| ENSCAFG00000012297 | 0.032157 | 0.064837 |
| ENSCAFG00000012635 | 0.014304 | 0.048595 |
| ENSCAFG00000012660 | 0.02224 | 0.056282 |
| ENSCAFG00000012867 | 0.000285 | 0.008845 |
| ENSCAFG00000012883 | 0.000946 | 0.015645 |
| ENSCAFG00000013100 | 0.065643 | 0.087525 |
| ENSCAFG00000013198 | 0.001503 | 0.017751 |
| ENSCAFG00000013218 | 0.004141 | 0.030203 |
| ENSCAFG00000013220 | 0.042616 | 0.072888 |
| ENSCAFG00000013239 | 0.079359 | 0.093275 |
| ENSCAFG00000013254 | 0.045794 | 0.075211 |
| ENSCAFG00000013299 | 0.060528 | 0.085776 |
| ENSCAFG00000013302 | 0.008493 | 0.038296 |
| ENSCAFG00000013316 | 0.044498 | 0.075071 |
| ENSCAFG00000013712 | 0.051436 | 0.078741 |
| ENSCAFG00000013717 | 0.0906 | 0.09942 |
| ENSCAFG00000013875 | 0.000763 | 0.01351 |
| ENSCAFG00000013883 | 0.058552 | 0.083453 |
| ENSCAFG00000013955 | 0.040599 | 0.074582 |
| ENSCAFG00000013999 | 0.006888 | 0.037134 |
| ENSCAFG00000014061 | 0.048753 | 0.076523 |
| ENSCAFG00000014129 | 0.002157 | 0.021395 |
| ENSCAFG00000014332 | 0.097468 | 0.100299 |
| ENSCAFG00000014339 | 0.098997 | 0.099397 |
| ENSCAFG00000014444 | 0.030478 | 0.062989 |
| ENSCAFG00000014522 | 0.006014 | 0.03551 |
| ENSCAFG00000014599 | 0.03058 | 0.062677 |
| ENSCAFG00000014674 | 0.000746 | 0.014223 |
| ENSCAFG00000014685 | 0.022786 | 0.057079 |
| ENSCAFG00000014774 | 0.097091 | 0.100327 |
| ENSCAFG00000014835 | 0.034169 | 0.065185 |
| ENSCAFG00000015003 | 0.048062 | 0.076406 |
| ENSCAFG00000015172 | 0.005787 | 0.035003 |
| ENSCAFG00000015197 | 0.015021 | 0.049015 |
| ENSCAFG00000015328 | 0.030284 | 0.063649 |
| ENSCAFG00000015337 | 0.025248 | 0.057445 |
| ENSCAFG00000015372 | 0.030349 | 0.063249 |
| ENSCAFG00000015495 | 0.076359 | 0.091928 |
| ENSCAFG00000015524 | 0.068734 | 0.089716 |
| ENSCAFG00000015622 | 0.006984 | 0.036849 |
| ENSCAFG00000015665 | 0.08441 | 0.095588 |
| ENSCAFG00000015718 | 0.077713 | 0.092214 |
| ENSCAFG00000015865 | 0.096408 | 0.10131 |
| ENSCAFG00000015892 | 0.005347 | 0.033999 |
| ENSCAFG00000015972 | 0.058146 | 0.083353 |
| ENSCAFG00000016096 | 0.018188 | 0.050683 |
| ENSCAFG00000016168 | 0.032396 | 0.064274 |
| ENSCAFG00000016184 | 0.065386 | 0.087652 |
| ENSCAFG00000016558 | 0.012424 | 0.046682 |
| ENSCAFG00000016570 | 0.004199 | 0.02975 |
| ENSCAFG00000016623 | 0.082252 | 0.094003 |
| ENSCAFG00000016787 | 0.023927 | 0.057057 |
| ENSCAFG00000016835 | 0.056016 | 0.081717 |
| ENSCAFG00000016878 | 0.095192 | 0.100887 |
| ENSCAFG00000016885 | 0.042455 | 0.073628 |
| ENSCAFG00000016943 | 0.072651 | 0.090997 |
| ENSCAFG00000016954 | 0.009922 | 0.041707 |
| ENSCAFG00000016989 | 0.001866 | 0.021039 |
| ENSCAFG00000017059 | 0.020894 | 0.055124 |
| ENSCAFG00000017108 | 0.074637 | 0.091634 |
| ENSCAFG00000017142 | 0.097516 | 0.099522 |
| ENSCAFG00000017144 | 2.49E-07 | 6.17E-05 |
| ENSCAFG00000017314 | 0.093246 | 0.100108 |
| ENSCAFG00000017344 | 0.08211 | 0.094274 |
| ENSCAFG00000017575 | 0.001377 | 0.01897 |
| ENSCAFG00000017616 | 0.008305 | 0.038863 |
| ENSCAFG00000017707 | 0.002394 | 0.021994 |
| ENSCAFG00000017764 | 0.070777 | 0.090947 |
| ENSCAFG00000017800 | 0.053104 | 0.080304 |
| ENSCAFG00000017907 | 0.064307 | 0.087627 |
| ENSCAFG00000017932 | 0.004697 | 0.03148 |
| ENSCAFG00000018024 | 0.085462 | 0.096339 |
| ENSCAFG00000018033 | 0.076681 | 0.091427 |
| ENSCAFG00000018038 | 0.044234 | 0.075137 |
| ENSCAFG00000018064 | 0.09871 | 0.099919 |
| ENSCAFG00000018082 | 0.021816 | 0.056357 |
| ENSCAFG00000018115 | 0.018181 | 0.051237 |
| ENSCAFG00000018266 | 0.098751 | 0.099554 |
| ENSCAFG00000018309 | 0.04667 | 0.074672 |
| ENSCAFG00000018493 | 0.015292 | 0.049251 |
| ENSCAFG00000018577 | 0.094466 | 0.100981 |
| ENSCAFG00000018725 | 0.000604 | 0.013616 |
| ENSCAFG00000018976 | 0.02721 | 0.060252 |
| ENSCAFG00000019051 | 0.032461 | 0.063892 |
| ENSCAFG00000019136 | 0.057418 | 0.082789 |
| ENSCAFG00000019176 | 0.067704 | 0.089312 |
| ENSCAFG00000019186 | 0.028461 | 0.062462 |
| ENSCAFG00000019344 | 0.010716 | 0.042863 |
| ENSCAFG00000019377 | 0.096553 | 0.101034 |
| ENSCAFG00000019438 | 0.086221 | 0.096319 |
| ENSCAFG00000019585 | 0.091568 | 0.0996 |
| ENSCAFG00000019771 | 0.048812 | 0.076134 |
| ENSCAFG00000019847 | 0.04208 | 0.073491 |
| ENSCAFG00000019871 | 0.033953 | 0.065273 |
| ENSCAFG00000019901 | 0.092449 | 0.099684 |
| ENSCAFG00000019992 | 5.55E-07 | 6.88E-05 |
| ENSCAFG00000020014 | 0.00782 | 0.038024 |
| ENSCAFG00000020029 | 0.045987 | 0.074541 |
| ENSCAFG00000020088 | 0.00238 | 0.022705 |
| ENSCAFG00000020208 | 0.041898 | 0.073693 |
| ENSCAFG00000020220 | 0.045419 | 0.075596 |
| ENSCAFG00000020224 | 0.00907 | 0.039462 |
| ENSCAFG00000020305 | 0.033058 | 0.064051 |
| ENSCAFG00000020318 | 0.009467 | 0.04048 |
| ENSCAFG00000020319 | 2.51E-06 | 0.000156 |
| ENSCAFG00000020361 | 0.045314 | 0.075931 |
| ENSCAFG00000023349 | 0.041771 | 0.073994 |
| ENSCAFG00000024647 | 0.0799 | 0.093029 |
| ENSCAFG00000025156 | 0.008679 | 0.038434 |
| ENSCAFG00000028614 | 0.071015 | 0.090783 |
| ENSCAFG00000028683 | 0.048497 | 0.076607 |
| ENSCAFG00000028770 | 0.08617 | 0.096698 |
| ENSCAFG00000028842 | 0.049452 | 0.076651 |
| ENSCAFG00000028968 | 0.026138 | 0.05893 |
| ENSCAFG00000029016 | 0.019636 | 0.052364 |
| ENSCAFG00000029027 | 0.056549 | 0.082013 |
| ENSCAFG00000029352 | 0.032334 | 0.064669 |
| ENSCAFG00000029613 | 0.064255 | 0.08804 |
| ENSCAFG00000029617 | 0.006624 | 0.038206 |
| ENSCAFG00000029728 | 0.070008 | 0.090427 |
| ENSCAFG00000029810 | 0.053916 | 0.081038 |
| ENSCAFG00000029976 | 0.081117 | 0.093567 |
| ENSCAFG00000030544 | 0.019383 | 0.05225 |
| ENSCAFG00000030599 | 0.0072 | 0.037202 |
| ENSCAFG00000031318 | 0.063595 | 0.088605 |
| ENSCAFG00000031331 | 0.012469 | 0.046154 |
| ENSCAFG00000031652 | 0.004117 | 0.030938 |
| ENSCAFG00000031849 | 0.055111 | 0.081354 |
| ENSCAFG00000032060 | 0.042536 | 0.073256 |
| ENSCAFG00000032251 | 0.011535 | 0.044697 |
| ENSCAFG00000032367 | 0.041533 | 0.074639 |
| ENSCAFG00000032423 | 0.073633 | 0.091304 |
| ENSCAFG00000032538 | 0.014668 | 0.049159 |
| ENSCAFG00000032597 | 0.074324 | 0.091703 |

**Table S5. Genes that show parallel evolution signals and parallel acid amino sites in each gene**

| dog gene id | position | branch 1 ancestor site | branch 2 ancestor site | branch 1/2 site |
| --- | --- | --- | --- | --- |
| ENSCAFG00000003189 | 141 | (V) | (V) | (I) |
| ENSCAFG00000005463 | 176 | (S) | (S) | (G) |
| ENSCAFG00000005485 | 291 | (S) | (S) | (P) |
| ENSCAFG00000006278 | 89 | (Y) | (Y) | (F) |
| ENSCAFG00000006389 | 25 | (L) | (L) | (I) |
| ENSCAFG00000006460 | 22 | (A) | (A) | (T) |
| ENSCAFG00000008681 | 267 | (E) | (E) | (Q) |
| ENSCAFG00000009087 | 231 | (K) | (K) | (R) |
| ENSCAFG00000009246 | 126 | (K) | (K) | (R) |
| ENSCAFG00000009246 | 69 | (T) | (T) | (M) |
| ENSCAFG00000014943 | 128 | (E) | (E) | (D) |
| ENSCAFG00000014943 | 308 | (A) | (A) | (G) |
| ENSCAFG00000015320 | 377 | (F) | (F) | (I) |
| ENSCAFG00000015320 | 5 | (A) | (A) | (V) |
| ENSCAFG00000015576 | 108 | (R) | (R) | (H) |
| ENSCAFG00000015622 | 83 | (G) | (G) | (R) |
| ENSCAFG00000018846 | 111 | (R) | (R) | (H) |
| ENSCAFG00000031613 | 118 | (T) | (T) | (S) |
| ENSCAFG00000001869 | 170 | (H) | (H) | (N) |
| ENSCAFG00000003623 | 23 | (V) | (V) | (L) |
| ENSCAFG00000004813 | 65 | (F) | (F) | (L) |
| ENSCAFG00000007277 | 14 | (C) | (C) | (S) |
| ENSCAFG00000007277 | 62 | (V) | (V) | (M) |
| ENSCAFG00000009467 | 111 | (L) | (L) | (V) |
| ENSCAFG00000009488 | 11 | (D) | (D) | (E) |
| ENSCAFG00000010404 | 323 | (A) | (A) | (V) |
| ENSCAFG00000011671 | 433 | (I) | (I) | (V) |
| ENSCAFG00000012068 | 12 | (T) | (T) | (A) |
| ENSCAFG00000017077 | 606 | (S) | (S) | (P) |
| ENSCAFG00000018725 | 4 | (V) | (V) | (L) |
| ENSCAFG00000018932 | 107 | (P) | (P) | (Q) |
| ENSCAFG00000029980 | 62 | (T) | (T) | (A) |
| ENSCAFG00000032587 | 23 | (T) | (T) | (S) |
| ENSCAFG00000000948 | 628 | (S) | (S) | (G) |
| ENSCAFG00000001075 | 437 | (H) | (H) | (N) |
| ENSCAFG00000005425 | 23 | (I) | (I) | (V) |
| ENSCAFG00000005491 | 73 | (I) | (I) | (L) |
| ENSCAFG00000009409 | 122 | (S) | (S) | (G) |
| ENSCAFG00000009698 | 22 | (T) | (T) | (A) |
| ENSCAFG00000010200 | 38 | (M) | (M) | (L) |
| ENSCAFG00000013425 | 103 | (R) | (R) | (K) |
| ENSCAFG00000013817 | 8 | (S) | (S) | (R) |
| ENSCAFG00000018314 | 4 | (C) | (C) | (Y) |
| ENSCAFG00000018822 | 5 | (A) | (A) | (T) |
| ENSCAFG00000030018 | 55 | (L) | (L) | (I) |
| ENSCAFG00000005936 | 328 | (T) | (T) | (A) |
| ENSCAFG00000006142 | 28 | (M) | (M) | (L) |
| ENSCAFG00000006995 | 644 | (S) | (S) | (N) |
| ENSCAFG00000006995 | 826 | (L) | (L) | (P) |
| ENSCAFG00000007749 | 47 | (T) | (T) | (S) |
| ENSCAFG00000007950 | 153 | (I) | (I) | (L) |
| ENSCAFG00000007967 | 301 | (G) | (G) | (R) |
| ENSCAFG00000008292 | 306 | (P) | (P) | (Q) |
| ENSCAFG00000008298 | 123 | (M) | (M) | (L) |
| ENSCAFG00000009517 | 382 | (V) | (V) | (F) |
| ENSCAFG00000010483 | 46 | (V) | (V) | (I) |
| ENSCAFG00000010532 | 189 | (E) | (E) | (A) |
| ENSCAFG00000015595 | 19 | (M) | (M) | (T) |
| ENSCAFG00000019730 | 300 | (R) | (R) | (H) |
| ENSCAFG00000023408 | 63 | (L) | (L) | (I) |
| ENSCAFG00000000642 | 102 | (E) | (E) | (D) |
| ENSCAFG00000003733 | 423 | (T) | (T) | (A) |
| ENSCAFG00000004068 | 133 | (P) | (P) | (A) |
| ENSCAFG00000004825 | 32 | (R) | (R) | (Q) |
| ENSCAFG00000004825 | 96 | (D) | (D) | (H) |
| ENSCAFG00000004888 | 13 | (Q) | (Q) | (K) |
| ENSCAFG00000005169 | 242 | (R) | (R) | (K) |
| ENSCAFG00000009303 | 7 | (I) | (I) | (V) |
| ENSCAFG00000009840 | 248 | (R) | (R) | (K) |
| ENSCAFG00000014014 | 185 | (S) | (S) | (N) |
| ENSCAFG00000023629 | 182 | (D) | (D) | (G) |
| ENSCAFG00000032367 | 32 | (I) | (I) | (V) |
| ENSCAFG00000032525 | 247 | (P) | (P) | (T) |
| ENSCAFG00000032525 | 264 | (S) | (S) | (P) |
| ENSCAFG00000009617 | 123 | (S) | (S) | (N) |
| ENSCAFG00000009617 | 255 | (F) | (F) | (L) |
| ENSCAFG00000011654 | 3 | (R) | (R) | (H) |
| ENSCAFG00000023997 | 24 | (R) | (R) | (Q) |
| ENSCAFG00000030054 | 72 | (Q) | (Q) | (H) |
| ENSCAFG00000002993 | 219 | (V) | (V) | (M) |
| ENSCAFG00000002993 | 68 | (P) | (P) | (T) |
| ENSCAFG00000004163 | 295 | (G) | (G) | (R) |
| ENSCAFG00000004373 | 179 | (M) | (M) | (I) |
| ENSCAFG00000004764 | 42 | (I) | (I) | (V) |
| ENSCAFG00000005655 | 12 | (E) | (E) | (D) |
| ENSCAFG00000006462 | 14 | (T) | (T) | (M) |
| ENSCAFG00000006579 | 191 | (A) | (A) | (E) |
| ENSCAFG00000008077 | 3 | (E) | (E) | (D) |
| ENSCAFG00000011030 | 57 | (R) | (R) | (K) |
| ENSCAFG00000016756 | 249 | (D) | (D) | (E) |
| ENSCAFG00000018022 | 111 | (I) | (I) | (L) |
| ENSCAFG00000018578 | 339 | (R) | (R) | (H) |
| ENSCAFG00000000412 | 110 | (N) | (N) | (K) |
| ENSCAFG00000000412 | 112 | (C) | (C) | (F) |
| ENSCAFG00000001043 | 65 | (R) | (R) | (H) |
| ENSCAFG00000001416 | 183 | (T) | (T) | (S) |
| ENSCAFG00000001416 | 282 | (R) | (R) | (Q) |
| ENSCAFG00000001767 | 45 | (T) | (T) | (I) |
| ENSCAFG00000002323 | 289 | (S) | (S) | (N) |
| ENSCAFG00000004648 | 24 | (L) | (L) | (F) |
| ENSCAFG00000010827 | 76 | (K) | (K) | (Q) |
| ENSCAFG00000011487 | 121 | (R) | (R) | (Q) |
| ENSCAFG00000013388 | 2 | (C) | (C) | (S) |
| ENSCAFG00000017129 | 47 | (I) | (I) | (V) |
| ENSCAFG00000019965 | 41 | (I) | (I) | (V) |
| ENSCAFG00000000226 | 237 | (G) | (G) | (S) |
| ENSCAFG00000000234 | 252 | (G) | (G) | (D) |
| ENSCAFG00000000314 | 229 | (D) | (D) | (E) |
| ENSCAFG00000000314 | 237 | (I) | (I) | (V) |
| ENSCAFG00000000314 | 28 | (P) | (P) | (L) |
| ENSCAFG00000004628 | 10 | (R) | (R) | (H) |
| ENSCAFG00000006757 | 65 | (E) | (E) | (D) |
| ENSCAFG00000007754 | 581 | (V) | (V) | (I) |
| ENSCAFG00000010238 | 652 | (S) | (S) | (N) |
| ENSCAFG00000011833 | 175 | (L) | (L) | (S) |
| ENSCAFG00000011948 | 110 | (L) | (L) | (Q) |
| ENSCAFG00000012108 | 306 | (S) | (S) | (N) |
| ENSCAFG00000013601 | 359 | (F) | (F) | (L) |
| ENSCAFG00000014220 | 679 | (R) | (R) | (K) |
| ENSCAFG00000014346 | 441 | (K) | (K) | (Q) |
| ENSCAFG00000014346 | 522 | (F) | (F) | (I) |
| ENSCAFG00000014346 | 524 | (D) | (D) | (E) |
| ENSCAFG00000015034 | 298 | (P) | (P) | (S) |
| ENSCAFG00000015143 | 261 | (L) | (L) | (V) |
| ENSCAFG00000015143 | 376 | (E) | (E) | (D) |
| ENSCAFG00000017062 | 158 | (S) | (S) | (R) |
| ENSCAFG00000031211 | 7 | (R) | (R) | (Q) |
| ENSCAFG00000001567 | 81 | (E) | (E) | (D) |
| ENSCAFG00000001951 | 295 | (A) | (A) | (V) |
| ENSCAFG00000002370 | 37 | (M) | (M) | (L) |
| ENSCAFG00000003154 | 6 | (F) | (F) | (L) |
| ENSCAFG00000003194 | 12 | (S) | (S) | (P) |
| ENSCAFG00000003477 | 92 | (R) | (R) | (K) |
| ENSCAFG00000006401 | 189 | (Q) | (Q) | (K) |
| ENSCAFG00000007486 | 258 | (S) | (S) | (N) |
| ENSCAFG00000012191 | 58 | (L) | (L) | (F) |
| ENSCAFG00000016623 | 121 | (N) | (N) | (D) |
| ENSCAFG00000018942 | 169 | (Q) | (Q) | (H) |
| ENSCAFG00000029011 | 89 | (S) | (S) | (T) |
| ENSCAFG00000032648 | 15 | (A) | (A) | (S) |
| ENSCAFG00000000028 | 135 | (R) | (R) | (G) |
| ENSCAFG00000000028 | 66 | (Y) | (Y) | (F) |
| ENSCAFG00000001798 | 297 | (P) | (P) | (S) |
| ENSCAFG00000002439 | 250 | (S) | (S) | (G) |
| ENSCAFG00000002439 | 288 | (C) | (C) | (G) |
| ENSCAFG00000003399 | 183 | (R) | (R) | (H) |
| ENSCAFG00000003613 | 258 | (S) | (S) | (G) |
| ENSCAFG00000008174 | 191 | (N) | (N) | (T) |
| ENSCAFG00000008389 | 152 | (P) | (P) | (L) |
| ENSCAFG00000009214 | 322 | (M) | (M) | (V) |
| ENSCAFG00000009230 | 49 | (R) | (R) | (Q) |
| ENSCAFG00000009323 | 111 | (S) | (S) | (N) |
| ENSCAFG00000010093 | 111 | (S) | (S) | (A) |
| ENSCAFG00000010093 | 121 | (I) | (I) | (V) |
| ENSCAFG00000010093 | 128 | (I) | (I) | (V) |
| ENSCAFG00000010093 | 133 | (T) | (T) | (S) |
| ENSCAFG00000010093 | 147 | (M) | (M) | (I) |
| ENSCAFG00000010093 | 151 | (M) | (M) | (L) |
| ENSCAFG00000010093 | 16 | (I) | (I) | (T) |
| ENSCAFG00000010093 | 166 | (T) | (T) | (S) |
| ENSCAFG00000010093 | 168 | (T) | (T) | (S) |
| ENSCAFG00000010093 | 169 | (D) | (D) | (E) |
| ENSCAFG00000010093 | 173 | (R) | (R) | (K) |
| ENSCAFG00000010093 | 178 | (S) | (S) | (A) |
| ENSCAFG00000010093 | 188 | (K) | (K) | (R) |
| ENSCAFG00000010093 | 228 | (M) | (M) | (L) |
| ENSCAFG00000010093 | 229 | (L) | (L) | (V) |
| ENSCAFG00000010093 | 23 | (K) | (K) | (Q) |
| ENSCAFG00000010093 | 232 | (V) | (V) | (L) |
| ENSCAFG00000010093 | 256 | (T) | (T) | (N) |
| ENSCAFG00000010093 | 260 | (I) | (I) | (L) |
| ENSCAFG00000010093 | 281 | (T) | (T) | (S) |
| ENSCAFG00000010093 | 282 | (R) | (R) | (K) |
| ENSCAFG00000010093 | 33 | (M) | (M) | (L) |
| ENSCAFG00000010093 | 343 | (N) | (N) | (H) |
| ENSCAFG00000010093 | 345 | (S) | (S) | (T) |
| ENSCAFG00000010093 | 357 | (A) | (A) | (S) |
| ENSCAFG00000010093 | 368 | (A) | (A) | (G) |
| ENSCAFG00000010093 | 369 | (I) | (I) | (V) |
| ENSCAFG00000010093 | 380 | (D) | (D) | (E) |
| ENSCAFG00000010093 | 384 | (T) | (T) | (M) |
| ENSCAFG00000010093 | 392 | (I) | (I) | (V) |
| ENSCAFG00000010093 | 398 | (D) | (D) | (E) |
| ENSCAFG00000010093 | 400 | (S) | (S) | (G) |
| ENSCAFG00000010093 | 44 | (I) | (I) | (V) |
| ENSCAFG00000010093 | 9 | (D) | (D) | (S) |
| ENSCAFG00000013333 | 500 | (I) | (I) | (V) |
| ENSCAFG00000015045 | 59 | (T) | (T) | (K) |
| ENSCAFG00000019111 | 119 | (S) | (S) | (A) |
| ENSCAFG00000031675 | 74 | (A) | (A) | (V) |
| ENSCAFG00000001343 | 175 | (R) | (R) | (H) |
| ENSCAFG00000001343 | 179 | (R) | (R) | (K) |
| ENSCAFG00000003322 | 38 | (M) | (M) | (L) |
| ENSCAFG00000005659 | 255 | (C) | (C) | (Y) |
| ENSCAFG00000009552 | 259 | (L) | (L) | (I) |
| ENSCAFG00000012361 | 185 | (I) | (I) | (M) |
| ENSCAFG00000015349 | 49 | (E) | (E) | (D) |
| ENSCAFG00000020105 | 112 | (Q) | (Q) | (K) |
| ENSCAFG00000028739 | 34 | (E) | (E) | (V) |
| ENSCAFG00000030033 | 145 | (Q) | (Q) | (H) |
| ENSCAFG00000031516 | 4 | (K) | (K) | (R) |
| ENSCAFG00000003235 | 118 | (T) | (T) | (A) |
| ENSCAFG00000004497 | 1 | (T) | (T) | (M) |
| ENSCAFG00000004549 | 138 | (T) | (T) | (I) |
| ENSCAFG00000004769 | 500 | (G) | (G) | (D) |
| ENSCAFG00000007753 | 72 | (E) | (E) | (K) |
| ENSCAFG00000009261 | 189 | (F) | (F) | (L) |
| ENSCAFG00000009304 | 340 | (F) | (F) | (Y) |
| ENSCAFG00000009579 | 10 | (Q) | (Q) | (P) |
| ENSCAFG00000010928 | 528 | (E) | (E) | (D) |
| ENSCAFG00000012941 | 160 | (Q) | (Q) | (K) |
| ENSCAFG00000013117 | 91 | (K) | (K) | (R) |
| ENSCAFG00000013211 | 62 | (Y) | (Y) | (H) |
| ENSCAFG00000015735 | 319 | (A) | (A) | (T) |
| ENSCAFG00000017154 | 164 | (L) | (L) | (M) |
| ENSCAFG00000017154 | 67 | (G) | (G) | (V) |
| ENSCAFG00000017532 | 325 | (P) | (P) | (A) |
| ENSCAFG00000023033 | 93 | (M) | (M) | (V) |
| ENSCAFG00000002091 | 161 | (Q) | (Q) | (H) |
| ENSCAFG00000007114 | 212 | (C) | (C) | (Y) |
| ENSCAFG00000009287 | 104 | (V) | (V) | (F) |
| ENSCAFG00000010089 | 54 | (K) | (K) | (R) |
| ENSCAFG00000011208 | 90 | (N) | (N) | (S) |
| ENSCAFG00000012735 | 22 | (I) | (I) | (V) |
| ENSCAFG00000013674 | 117 | (T) | (T) | (S) |
| ENSCAFG00000015537 | 83 | (R) | (R) | (K) |
| ENSCAFG00000015551 | 17 | (M) | (M) | (I) |
| ENSCAFG00000016035 | 58 | (P) | (P) | (H) |
| ENSCAFG00000017076 | 62 | (R) | (R) | (Q) |
| ENSCAFG00000017656 | 100 | (T) | (T) | (M) |
| ENSCAFG00000017656 | 98 | (I) | (I) | (V) |
| ENSCAFG00000018647 | 71 | (S) | (S) | (N) |
| ENSCAFG00000029496 | 28 | (L) | (L) | (F) |
| ENSCAFG00000002658 | 12 | (G) | (G) | (D) |
| ENSCAFG00000007682 | 202 | (K) | (K) | (R) |
| ENSCAFG00000007779 | 103 | (S) | (S) | (G) |
| ENSCAFG00000007779 | 104 | (I) | (I) | (V) |
| ENSCAFG00000007779 | 111 | (Y) | (Y) | (H) |
| ENSCAFG00000007789 | 108 | (L) | (L) | (I) |
| ENSCAFG00000007789 | 83 | (L) | (L) | (F) |
| ENSCAFG00000008324 | 173 | (N) | (N) | (K) |
| ENSCAFG00000010004 | 3 | (E) | (E) | (D) |
| ENSCAFG00000013457 | 17 | (R) | (R) | (H) |
| ENSCAFG00000017708 | 117 | (A) | (A) | (T) |
| ENSCAFG00000017708 | 231 | (Q) | (Q) | (K) |
| ENSCAFG00000017708 | 288 | (I) | (I) | (V) |
| ENSCAFG00000017708 | 7 | (F) | (F) | (L) |
| ENSCAFG00000030877 | 145 | (P) | (P) | (Q) |
| ENSCAFG00000000310 | 16 | (R) | (R) | (H) |
| ENSCAFG00000002129 | 110 | (Y) | (Y) | (F) |
| ENSCAFG00000003315 | 168 | (V) | (V) | (A) |
| ENSCAFG00000004647 | 256 | (Q) | (Q) | (H) |
| ENSCAFG00000005022 | 56 | (L) | (L) | (P) |
| ENSCAFG00000006001 | 185 | (N) | (N) | (S) |
| ENSCAFG00000007160 | 107 | (V) | (V) | (A) |
| ENSCAFG00000007160 | 12 | (D) | (D) | (E) |
| ENSCAFG00000007160 | 164 | (T) | (T) | (A) |
| ENSCAFG00000007731 | 25 | (T) | (T) | (M) |
| ENSCAFG00000010731 | 17 | (V) | (V) | (I) |
| ENSCAFG00000012286 | 251 | (V) | (V) | (I) |
| ENSCAFG00000012423 | 99 | (N) | (N) | (D) |
| ENSCAFG00000012539 | 26 | (C) | (C) | (S) |
| ENSCAFG00000012913 | 59 | (I) | (I) | (V) |
| ENSCAFG00000019835 | 142 | (G) | (G) | (E) |
| ENSCAFG00000020030 | 223 | (Y) | (Y) | (F) |
| ENSCAFG00000020303 | 357 | (Y) | (Y) | (F) |
| ENSCAFG00000020305 | 430 | (R) | (R) | (K) |
| ENSCAFG00000030579 | 134 | (T) | (T) | (A) |
| ENSCAFG00000031021 | 25 | (V) | (V) | (I) |
| ENSCAFG00000032209 | 72 | (D) | (D) | (E) |
| ENSCAFG00000002770 | 135 | (S) | (S) | (G) |
| ENSCAFG00000003175 | 227 | (V) | (V) | (G) |
| ENSCAFG00000003984 | 97 | (I) | (I) | (V) |
| ENSCAFG00000004023 | 342 | (P) | (P) | (S) |
| ENSCAFG00000005587 | 62 | (R) | (R) | (H) |
| ENSCAFG00000005690 | 68 | (R) | (R) | (H) |
| ENSCAFG00000005738 | 344 | (F) | (F) | (L) |
| ENSCAFG00000007219 | 357 | (Q) | (Q) | (P) |
| ENSCAFG00000008747 | 18 | (M) | (M) | (L) |
| ENSCAFG00000009500 | 27 | (V) | (V) | (G) |
| ENSCAFG00000014390 | 195 | (S) | (S) | (A) |
| ENSCAFG00000014390 | 362 | (L) | (L) | (F) |
| ENSCAFG00000015343 | 378 | (L) | (L) | (S) |
| ENSCAFG00000018068 | 59 | (R) | (R) | (H) |
| ENSCAFG00000019717 | 6 | (A) | (A) | (V) |
| ENSCAFG00000029750 | 51 | (P) | (P) | (S) |
| ENSCAFG00000000373 | 160 | (I) | (I) | (V) |
| ENSCAFG00000000398 | 159 | (R) | (R) | (G) |
| ENSCAFG00000004072 | 127 | (L) | (L) | (S) |
| ENSCAFG00000004135 | 24 | (A) | (A) | (D) |
| ENSCAFG00000004212 | 259 | (S) | (S) | (N) |
| ENSCAFG00000010696 | 210 | (H) | (H) | (Q) |
| ENSCAFG00000011896 | 112 | (R) | (R) | (Q) |
| ENSCAFG00000011993 | 118 | (H) | (H) | (Q) |
| ENSCAFG00000011993 | 661 | (N) | (N) | (T) |
| ENSCAFG00000012086 | 259 | (R) | (R) | (K) |
| ENSCAFG00000012400 | 10 | (K) | (K) | (R) |
| ENSCAFG00000015233 | 148 | (T) | (T) | (N) |
| ENSCAFG00000016981 | 158 | (R) | (R) | (H) |
| ENSCAFG00000017421 | 14 | (S) | (S) | (T) |
| ENSCAFG00000018870 | 115 | (T) | (T) | (A) |
| ENSCAFG00000000067 | 145 | (F) | (F) | (Y) |
| ENSCAFG00000000079 | 81 | (R) | (R) | (K) |
| ENSCAFG00000004501 | 218 | (K) | (K) | (R) |
| ENSCAFG00000004501 | 249 | (K) | (K) | (Q) |
| ENSCAFG00000004932 | 72 | (I) | (I) | (V) |
| ENSCAFG00000007907 | 842 | (M) | (M) | (V) |
| ENSCAFG00000008407 | 290 | (Y) | (Y) | (F) |
| ENSCAFG00000013522 | 47 | (I) | (I) | (V) |
| ENSCAFG00000014008 | 145 | (M) | (M) | (V) |
| ENSCAFG00000014431 | 122 | (A) | (A) | (S) |
| ENSCAFG00000017337 | 53 | (C) | (C) | (S) |
| ENSCAFG00000017415 | 166 | (I) | (I) | (V) |
| ENSCAFG00000017877 | 204 | (Q) | (Q) | (R) |
| ENSCAFG00000020326 | 489 | (P) | (P) | (S) |
| ENSCAFG00000029733 | 114 | (Y) | (Y) | (H) |
| ENSCAFG00000030884 | 57 | (R) | (R) | (L) |
| ENSCAFG00000000927 | 71 | (R) | (R) | (L) |
| ENSCAFG00000002037 | 108 | (L) | (L) | (I) |
| ENSCAFG00000002069 | 302 | (E) | (E) | (D) |
| ENSCAFG00000005961 | 84 | (G) | (G) | (E) |
| ENSCAFG00000006791 | 126 | (L) | (L) | (P) |
| ENSCAFG00000006791 | 246 | (I) | (I) | (V) |
| ENSCAFG00000007007 | 757 | (Q) | (Q) | (P) |
| ENSCAFG00000007250 | 121 | (N) | (N) | (S) |
| ENSCAFG00000007250 | 122 | (V) | (V) | (I) |
| ENSCAFG00000007288 | 1 | (I) | (I) | (V) |
| ENSCAFG00000007954 | 32 | (M) | (M) | (V) |
| ENSCAFG00000012591 | 45 | (I) | (I) | (V) |
| ENSCAFG00000014674 | 147 | (R) | (R) | (H) |
| ENSCAFG00000014909 | 184 | (H) | (H) | (N) |
| ENSCAFG00000016117 | 79 | (V) | (V) | (G) |
| ENSCAFG00000016285 | 182 | (Q) | (Q) | (H) |
| ENSCAFG00000016293 | 73 | (M) | (M) | (T) |
| ENSCAFG00000016353 | 310 | (T) | (T) | (A) |
| ENSCAFG00000032423 | 30 | (Q) | (Q) | (E) |
| ENSCAFG00000004166 | 243 | (S) | (S) | (T) |
| ENSCAFG00000005031 | 365 | (V) | (V) | (I) |
| ENSCAFG00000005383 | 249 | (R) | (R) | (Q) |
| ENSCAFG00000005383 | 353 | (C) | (C) | (Y) |
| ENSCAFG00000005461 | 228 | (E) | (E) | (D) |
| ENSCAFG00000010436 | 6 | (T) | (T) | (I) |
| ENSCAFG00000011552 | 273 | (S) | (S) | (N) |
| ENSCAFG00000012176 | 8 | (M) | (M) | (V) |
| ENSCAFG00000012413 | 400 | (L) | (L) | (S) |
| ENSCAFG00000013751 | 82 | (Q) | (Q) | (K) |
| ENSCAFG00000013894 | 178 | (L) | (L) | (F) |
| ENSCAFG00000013894 | 216 | (H) | (H) | (Y) |
| ENSCAFG00000015665 | 353 | (P) | (P) | (T) |
| ENSCAFG00000023327 | 115 | (L) | (L) | (H) |
| ENSCAFG00000030810 | 96 | (C) | (C) | (Y) |
| ENSCAFG00000000856 | 54 | (S) | (S) | (G) |
| ENSCAFG00000000856 | 78 | (S) | (S) | (G) |
| ENSCAFG00000001008 | 140 | (R) | (R) | (H) |
| ENSCAFG00000004247 | 50 | (V) | (V) | (M) |
| ENSCAFG00000005838 | 186 | (I) | (I) | (V) |
| ENSCAFG00000006376 | 142 | (S) | (S) | (P) |
| ENSCAFG00000006376 | 48 | (I) | (I) | (V) |
| ENSCAFG00000006376 | 67 | (K) | (K) | (R) |
| ENSCAFG00000007767 | 362 | (S) | (S) | (N) |
| ENSCAFG00000009477 | 334 | (T) | (T) | (A) |
| ENSCAFG00000010111 | 44 | (E) | (E) | (D) |
| ENSCAFG00000011432 | 140 | (T) | (T) | (S) |
| ENSCAFG00000011663 | 48 | (L) | (L) | (I) |
| ENSCAFG00000014458 | 147 | (S) | (S) | (I) |
| ENSCAFG00000016376 | 224 | (R) | (R) | (K) |
| ENSCAFG00000016878 | 247 | (L) | (L) | (M) |
| ENSCAFG00000019772 | 55 | (V) | (V) | (I) |
| ENSCAFG00000031580 | 50 | (I) | (I) | (V) |
| ENSCAFG00000002664 | 412 | (R) | (R) | (K) |
| ENSCAFG00000002692 | 23 | (Y) | (Y) | (H) |
| ENSCAFG00000002721 | 307 | (M) | (M) | (T) |
| ENSCAFG00000005997 | 239 | (T) | (T) | (I) |
| ENSCAFG00000006089 | 86 | (E) | (E) | (D) |
| ENSCAFG00000007898 | 174 | (G) | (G) | (E) |
| ENSCAFG00000007948 | 161 | (H) | (H) | (Q) |
| ENSCAFG00000007948 | 98 | (H) | (H) | (Q) |
| ENSCAFG00000008087 | 194 | (G) | (G) | (R) |
| ENSCAFG00000008404 | 315 | (T) | (T) | (S) |
| ENSCAFG00000009207 | 299 | (S) | (S) | (A) |
| ENSCAFG00000010887 | 95 | (R) | (R) | (Q) |
| ENSCAFG00000016186 | 260 | (S) | (S) | (G) |
| ENSCAFG00000016204 | 344 | (I) | (I) | (V) |
| ENSCAFG00000016645 | 94 | (V) | (V) | (I) |
| ENSCAFG00000016978 | 35 | (R) | (R) | (K) |
| ENSCAFG00000031124 | 181 | (C) | (C) | (Y) |
| ENSCAFG00000004180 | 238 | (S) | (S) | (P) |
| ENSCAFG00000004876 | 224 | (K) | (K) | (R) |
| ENSCAFG00000006178 | 578 | (N) | (N) | (K) |
| ENSCAFG00000006790 | 276 | (C) | (C) | (R) |
| ENSCAFG00000007662 | 247 | (I) | (I) | (L) |
| ENSCAFG00000008376 | 51 | (C) | (C) | (S) |
| ENSCAFG00000009768 | 150 | (E) | (E) | (D) |
| ENSCAFG00000010511 | 131 | (C) | (C) | (Y) |
| ENSCAFG00000010835 | 105 | (E) | (E) | (D) |
| ENSCAFG00000010835 | 50 | (T) | (T) | (A) |
| ENSCAFG00000010835 | 9 | (V) | (V) | (A) |
| ENSCAFG00000010839 | 56 | (S) | (S) | (A) |
| ENSCAFG00000010952 | 225 | (T) | (T) | (A) |
| ENSCAFG00000010952 | 547 | (P) | (P) | (S) |
| ENSCAFG00000010952 | 581 | (I) | (I) | (V) |
| ENSCAFG00000012130 | 47 | (R) | (R) | (K) |
| ENSCAFG00000015188 | 89 | (L) | (L) | (P) |
| ENSCAFG00000015925 | 23 | (L) | (L) | (I) |
| ENSCAFG00000016158 | 195 | (M) | (M) | (T) |
| ENSCAFG00000016158 | 247 | (I) | (I) | (V) |
| ENSCAFG00000019064 | 214 | (L) | (L) | (F) |
| ENSCAFG00000025192 | 122 | (A) | (A) | (V) |
| ENSCAFG00000025192 | 78 | (S) | (S) | (C) |
